# Influence of soil depth, irrigation, and plant genotype on the soil microbiome, metaphenome, and carbon chemistry

**DOI:** 10.1128/mbio.01758-23

**Published:** 2023-09-20

**Authors:** Katherine I. Naasko, Daniel Naylor, Emily B. Graham, Sneha P. Couvillion, Robert Danczak, Nikola Tolic, Carrie Nicora, Steven Fransen, Haiying Tao, Kirsten S. Hofmockel, Janet K. Jansson

**Affiliations:** 1 Earth and Biological Sciences Directorate, Pacific Northwest National Laboratory, Richland, Washington, USA; 2 Department of Crop and Soil Sciences, Washington State University, Pullman, Washington, USA; 3 Department of Crop and Soil Sciences, Washington State University, Prosser, Washington, USA; 4 Department of Plant Science and Landscape Architecture, University of Connecticut, Storrs, Connecticut, USA; 5 Department of Agronomy, Iowa State University, Ames, Iowa, USA; University of Washington, Seattle, Washington, USA

**Keywords:** soil depth, multi-omics, calcareous soil, carbon storage, metaphenome, soil microbiome, carbon chemistry

## Abstract

Climate change is causing an increase in drought in many soil ecosystems and a loss of soil organic carbon. Calcareous soils may partially mitigate these losses via carbon capture and storage. Here, we aimed to determine how irrigation-supplied soil moisture and perennial plants impact biotic and abiotic soil properties that underpin deep soil carbon chemistry in an unfertilized calcareous soil. Soil was sampled up to 1 m in depth from irrigated and planted field treatments and was analyzed using a suite of omics and chemical analyses. The soil microbial community composition was impacted more by irrigation and plant cover treatments than by soil depth. By contrast, metabolomes, lipidomes, and proteomes differed more with soil depth than treatments. Deep soil (>50 cm) had higher soil pH and calcium concentrations and higher levels of organic acids, bicarbonate, and triacylglycerides. By contrast, surface soil (0–5 cm) had higher concentrations of soil organic matter, organic carbon, oxidizable carbon, and total nitrogen. Surface soils also had higher amounts of sugars, sugar alcohols, phosphocholines, and proteins that reflect osmotic and oxidative stress responses. The lipidome was more responsive to perennial tall wheatgrass treatments compared to the metabolome or proteome, with a striking change in diacylglyceride composition. Permanganate oxidizable carbon was more consistently correlated to metabolites and proteins than soil organic and inorganic carbon and soil organic matter. This study reveals specific compounds that reflect differences in organic, inorganic, and oxidizable soil carbon fractions that are impacted by interactions between irrigation-supplied moisture and plant cover in calcareous soil profiles.

**IMPORTANCE:**

Carbon is cycled through the air, plants, and belowground environment. Understanding soil carbon cycling in deep soil profiles will be important to mitigate climate change. Soil carbon cycling is impacted by water, plants, and soil microorganisms, in addition to soil mineralogy. Measuring biotic and abiotic soil properties provides a perspective of how soil microorganisms interact with the surrounding chemical environment. This study emphasizes the importance of considering biotic interactions with inorganic and oxidizable soil carbon in addition to total organic carbon in carbonate-containing soils for better informing soil carbon management decisions.

## INTRODUCTION

Soil represents an enormous global reservoir of terrestrial carbon (C) with 2,500 gigatons (Gt) of C in organic and inorganic forms to greater than 2 m in depth (1). The soil organic C (SOC) in the surface 20 cm of the soil profile represents ~750 Gt, which is approximately 30% of the total soil C pool (1, 2). Most SOC is stored in soil organic matter (SOM), which is essential to ecosystem processes that support terrestrial life on Earth (3, 4). The remaining 70% of terrestrial C is stored deeper than 20 cm in the soil profile (1, 2), which makes studying the fate of deep soil C imperative (3, 4). In addition to SOC, soil C can also be in the form of soil inorganic C (SIC), as carbonates that typically accumulate at depth in arid regions (2, 5). In total, SIC constitutes a third of the global C pool (1, 2). Specifically, calcareous soils, or those that are rich in calcium carbonate (CaCO_3_), cover nearly half of the Earth’s surface and constitute approximately 9 billion hectares of arable land worldwide (5, 6). In soils rich in inorganic carbon, both SOC and SIC are projected to decrease according to climate models (7 – 9). Climate change is also resulting in increased drought and elevated atmospheric carbon dioxide (CO_2_) levels (2, 5, 9), and the latter can cause SIC loss in calcareous soils (10). While non-carbonate-containing soils have the potential to partially mitigate increasing CO_2_ levels by storing C belowground in SOM (11), calcareous soils hold additional potential to store mineral C in CaCO_3_ (12). However, most previous soil C sequestration efforts have largely focused on organic C (9), and carbonate-containing soils have received much less attention as an additional reservoir for atmospheric CO_2_ removal.

Both abiotic and biotic soil properties can impact the fate and retention of soil C in calcareous soils. The main abiotic properties that impact SIC levels include soil pH (2, 7, 9), electrical conductivity (7), mean annual temperature (2), and land use (2, 9, 12). In saline and alkaline grassland soils, land degradation reduces SIC to a greater extent than SOC (7). Additionally, irrigation of high pH soils causes acidification and changes in redox potential (13). Furthermore, acidification is associated with loss of SIC via leaching of bicarbonate (9) and SOC via microbial respiration (13). However, soils with high Ca^+2^ concentrations may have a lower potential for SOM decomposition as higher Ca^+2^ concentrations are associated with lower SOC leaching losses, photooxidation, and CO_2_ respiration (14). Furthermore, exchangeable Ca^+2^ improves structural stability through cation bridges, and the presence of CaCO_3_ increases aggregate stability that traps SOC (14).

Biotic drivers of soil C cycling and retention begin with plant inputs and involve myriad organisms that live in the soil. Soil microorganisms are widely recognized as the main drivers of decomposition due to their metabolic diversity (15). The capacity of the microbiome to cycle C and decompose SOM is affected by resource availability that can vary through the soil profile (16 – 18). The turnover and distribution of bioavailable C with soil depth are impacted by factors such as mechanical disturbance (3), bioturbation by macrofauna (3), root chemistry (17), rhizodeposition (18, 19), and microbial turnover (16, 17). In particular, deep roots of perennial grasses impact translocation of new roots, litter, detritus, and biomass into the soil profile and thereby influence stability of deep soil C (18, 20). In fact, plant litter contributes to more efficient formation of particulate and mineral-associated SOM in subsoils compared to surface soils (21). Furthermore, the presence of plants influences the chemistry of mineral-associated SOM and increases the diversity of C compounds when compared to soils without plants (22). In surface soils and arable soils, plant rhizodeposition can stimulate C loss via microbial decomposition and subsequent CO_2_ release (19). However, the potential for C storage in deeper soils, marginal soils, and calcareous soils may be higher due to lower microbial activity associated with less available C and other nutrients (17, 18) and higher potential for necromass accumulation (18) and CaCO_3_ precipitation.

Most field research on microbial cycling of SOM has focused on the top 20 cm of the soil profile (23), and less is known about deeper soil layers and carbonate-containing soils. In addition, current understanding of plant, microbial, and mineral interactions that drive C cycling depends on loosely defined metabolic processes that are easily measured, such as soil respiration (17, 24, 25) and the formation of different SOM fractions (22, 23). Only a handful of studies have examined in-field relationships between soil microbial communities and C turnover, such as through C-labeling experiments in deep soils (18) and multi-omics analysis of field-sampled soils (26 – 29). Thus, a current knowledge gap is how the soil metaphenome (17) or combined phenotypes of the resident soil microbes, including their exchange of metabolites, production of extracellular proteins, and shifts in lipids, are influenced by soil depth in the field. Furthermore, how these biotic soil properties interact with soil moisture and other abiotic soil properties, including soil nitrogen (N) and the presence of calcium minerals that ultimately may influence the types and amounts of soil C that are present in deep calcareous soils, is unknown. Linking omics approaches with more traditional soil biogeochemistry measurements has promise to further our understanding of how soil microbes contribute to SOM transformations and how they drive soil C dynamics (30).

To address these knowledge gaps, we tested the effects of soil depth and moisture on plant-microbe-soil interactions using soil samples from an unfertilized, calcareous field soil, with and without irrigation and deep-rooted perennial grasses. Root biomass was observed as deep as 1 m. Soil cores from 0 to 1 m were collected in July 2020 from an arid field experiment established in 2018 at the Washington State University Irrigated Agricultural Research and Extension Center (IAREC) in Prosser, WA, USA. The soil cores were sampled 1 week following aboveground biomass harvest of the perennial tall wheatgrass plants and termination of irrigation. Therefore, plant and irrigation effects should be interpreted as legacy effects from the growing season. Four treatments were sampled from this field trial: unirrigated bare soil, irrigated bare soil, and two irrigated perennial tall wheatgrass (*Thinopyrum ponticum*) cultivars, Alkar and Jose. Perennial tall wheatgrass is a promising bioenergy crop that can form deep roots in marginal saline and alkaline soils (31, 32), such as the field site’s calcareous soils.

A combination of soil biotic and abiotic variables was determined to test for irrigation, cultivar, and depth effects on the soil microbiome and metaphenome. Amplicon sequencing was coupled with a multi-omics approach to characterize the soil microbiome composition, diversity, metabolome, lipidome, and proteome (33). These data were tested for correlations with several abiotic soil properties, including soil moisture, soil pH, total soil N (TN), and calcium (Ca) concentrations. In addition, different forms of soil C were determined, including SOM, total organic carbon (TOC), SIC, and permanganate oxidizable carbon (POXC). The POXC pool is an oxidizable fraction of SOC (34) that is suggested to indicate SOM stabilizing practices (35). Recently, POXC was shown to correspond to the bacterial metagenome more than other soil biological health metrics (36). We hypothesized that (i) irrigation lowers soil pH and levels of TOC and SIC through the soil profile; (ii) under irrigation, levels of TOC, SIC, and diversity of metabolic products are increased in the presence of the roots of perennial grasses as compared to bare soil; (iii) in deeper soil layers with low C and N concentrations, plant inputs stimulate the accumulation of SOC compared to surface soils; and (iv) the metaphenome in surface and deep soils is impacted by differences in abiotic parameters, including soil pH and Ca and other nutrient concentrations that reflect distinct differences in C metabolism at different soil depths. This rich data set highlights soil properties that reflect the plant-microbe-soil interactions that influence differences in stable and reactive soil C in deep calcareous soils.

## RESULTS AND DISCUSSION

### Soil organic and inorganic carbon pools differ with soil depth and irrigation more than plant treatments

**FIG 1:**
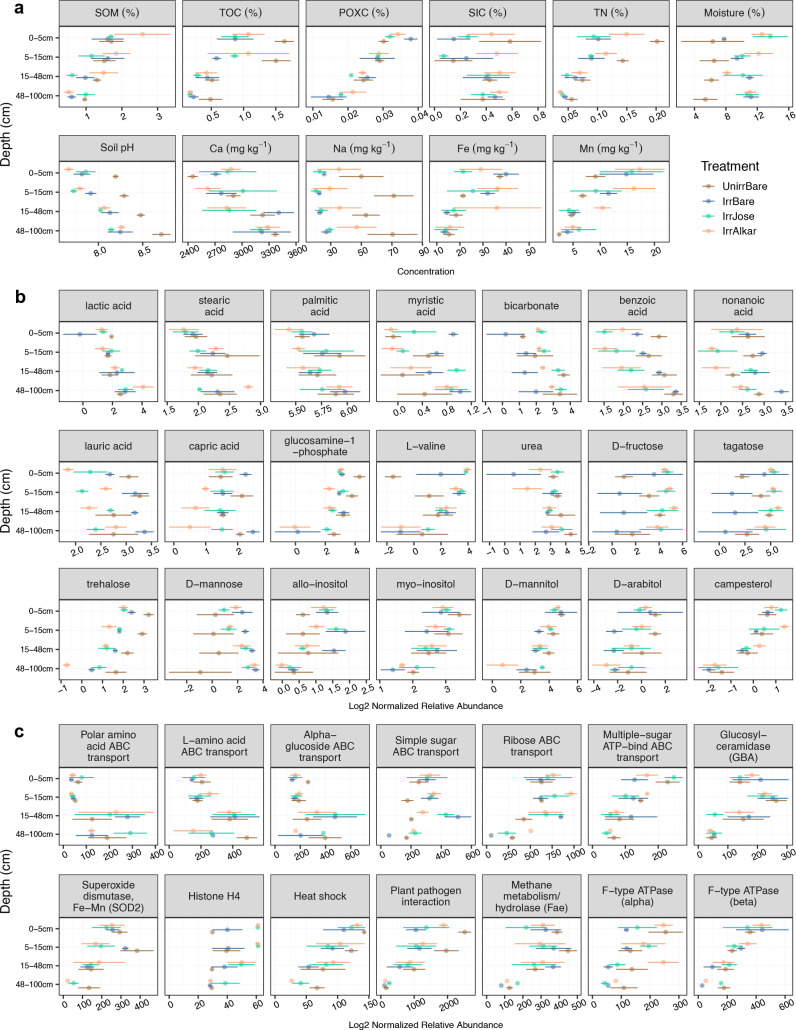
Soil depth and field treatment effects on (a) abiotic soil properties and log2 normalized relative abundances of (b) polar metabolites and (c) proteins. A single soil core was sampled from three replicate blocks of four treatments (i.e., unirrigated bare [brown] and three irrigated treatments including bare soil [blue], Alkar cultivar [orange], and Jose cultivar [green]) and separated into four depth increments (0–5, 5–15, 15–48, and 48–100 cm).

The plant-microbe-soil interactions that drive the accumulation of different forms of soil C varied with soil depth. Averaging across all treatments, SOM and organic pools of soil C were significantly higher in concentration on the soil surface (0–5 cm) compared to the deep soil layer (48–100 cm), including levels of SOM (1.90%–0.77%), TOC (1.11%–0.26%), and POXC (0.03%–0.02%) (all *P* < 0.01) (Fig. 1a; Table 1; Table S1). Concentrations of TN, Olsen (37) phosphorus (P), and metal micronutrients including iron (Fe) and manganese (Mn) were also significantly higher at the surface and decreased with depth (0.14%–0.04% TN, 30- to 12-ppm P; 32- to 15-ppm Fe^+2^; 14- to 4-ppm Mn^+2^) (all *P* < 0.01) (Fig. 1a; Table 1; Table S1). Accumulation of SOM, SOC, and other nutrients at the surface reflects enhanced plant and microbial activity and greater accumulation of aboveground plant biomass at the surface (3, 4). On the other hand, the deep soil horizon had higher concentrations of exchangeable calcium (2,719 to 3,269 ppm Ca^+2^; *P* < 0.01) and SIC (0.38% to 0.48%; *P* = 0.17) compared to the surface soil horizon, congruent with increased soil pH with depth (7.88–8.34, *P* < 0.01) (Fig. 1a; Table 1; Table S1). The significant increases in Ca concentrations and soil pH with depth are characteristic of arid soils with calcareous soil horizons (5, 6). While the increase in SIC with soil depth was not statistically significant, the SIC pool was nearly twice as large as the TOC pool in the deep soil horizon. Soil C cycling in surface soils with higher TOC and lower soil pH may be more impacted by TOC-Fe/Mn interactions, in contrast to the calcareous soil horizon with higher SIC and Ca. These findings emphasize that managing marginal lands with calcareous soils for enhanced C accumulation should leverage native conditions of greater TOC at the soil surface and SIC at all depths.

**TABLE 1:** Significant depth and treatment effects on abiotic soil properties as well as metabolites and proteins identified using multi-omics technologies^*a*^

Test	Property	Depth	Irrigation	Alkar	Jose
Abiotic	Organic carbon	−	−		
	Total nitrogen	−	−		
	Oxidizable carbon	−			
	Olsen phosphorus	−			
	Mn, Fe	−			
	Ca	+			
	Soil pH	+	−		
	Na		−		
Metabolite	Bicarbonate	+	−	+	+
	Benzoic acid	+		+	+
	Lauric acid			+	+
	Nonanoic acid			+	+
	Lactic acid	+			
	Myristic acid	+			
	Palmitic acid	+			
	Stearic acid	+			
	Arachidic acid	−			
	Linoleic acid	−			
	N-acetyl-D-mannosamine	−			
	Glucosamine-1-phosphate	−			
	L-valine	−			
	Urea		−		
	Trehalose	−	−		
	D-mannose		+		
	D-fructose			+	+
	Tagatose			+	+
	D-arabitol	−			
	D-mannitol	−			
	Campesterol	−			
	Allo-inositol	−			
	Myo-inositol	−			
Protein	Polar amino acid ABC transport	+			
	L-amino acid ABC transport	+			
	Alpha-glucoside ABC transport	+			
	Multiple sugar ATP-bind ABC transport	−			
	Glucosylceramidase	−			
	Superoxide dismutase, Fe-Mn (SOD2)	−			
	F-type ATPase	−		+	
	Ribose ABC transport	−			
	Histone H4	−		+	+
	Plant pathogen elongation/interaction	−			
	Heat shock	−			
	Methane metabolism/hydrolase (Fae)	−			

^*a*^ Properties denoted with plus or minus signs increased or decreased, respectively, over specified factor.

Irrigation also affected abiotic soil properties that impact C gains and losses in biomass production systems. As soil moisture content increased with irrigation, irrigated bare soils also had significantly lower soil pH and concentrations of TOC, TN, and sodium (Na) compared to unirrigated bare soils (all *P* < 0.01) (Fig. 1a; Table 1; Table S1). Irrigation of calcareous soils induces acidification, leaching, and mineralization via microbial CO_2_ respiration (7, 9, 13, 14). Additionally, water molecules react with CO_2_ to generate bicarbonate ions (HCO_3_^−^), which can leach deeper into the soil profile in weak associations with base cations, such as Na and Ca. However, under high Ca conditions, CO_2_ can react with Ca in abiotic or microbial CaCO_3_ precipitation (38). The effect of irrigation on TOC and TN loss was especially pronounced at the soil surface (Fig. 1a), which suggests that larger and more frequent fluctuations in soil moisture from surface drip irrigation stimulate microbial decomposition and CO_2_ respiration to a greater extent in surface soils than deep soils. Thus, in deep calcareous soil, horizons with smaller and less frequent fluctuations in soil moisture, CaCO_3_ precipitation may dominate with higher soil pH and Ca concentrations compared to surface soils. These results point to several microbial interactions with soil C and water that differ with depth, depending on soil pH and mineralogy.

### Soil metabolites differ more with soil depth than with field irrigation or plant treatments

**FIG 2:**
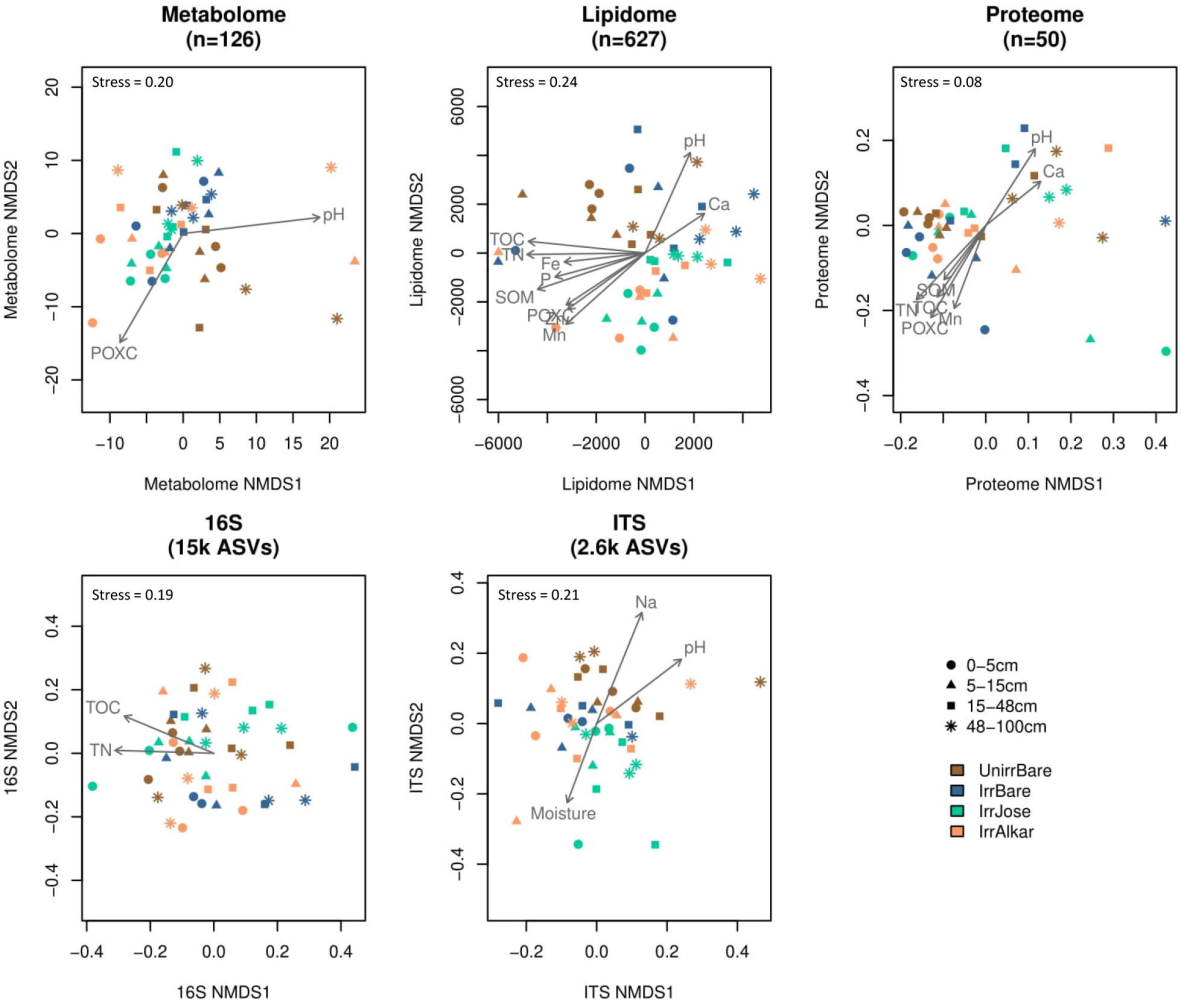
Multi-omics differences with soil depth and treatment. Dissimilarities were visualized using NMDS and were tested for correlations with abiotic soil properties and differences with soil depth and field treatment. A single soil core was sampled from three replicate blocks of four treatments (i.e., unirrigated bare [brown], irrigated bare soil [blue], irrigated Alkar [orange], and irrigated Jose [green]) and separated into four depth increments: 0–5 cm (circles), 5–15 cm (triangles), 15–48 cm (squares), and 48–100 cm (stars) to produce 48 soil samples.

**FIG 3:**
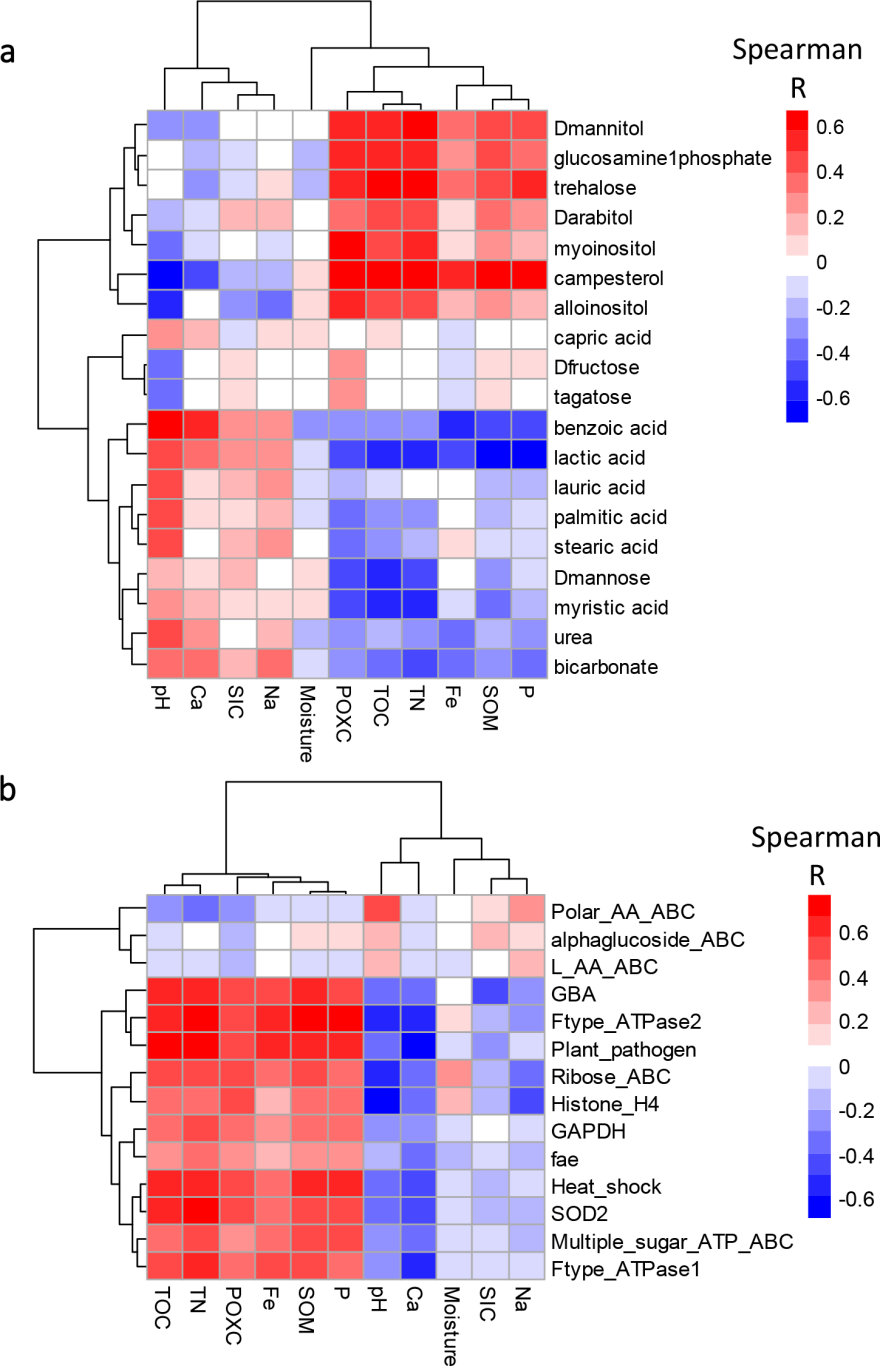
Spearman correlation heatmap of abiotic soil properties (absolute data) vs (a) log2 normalized polar metabolites and (b) log2 normalized proteins averaged across all field treatments and soil depth increments.

**FIG 4:**
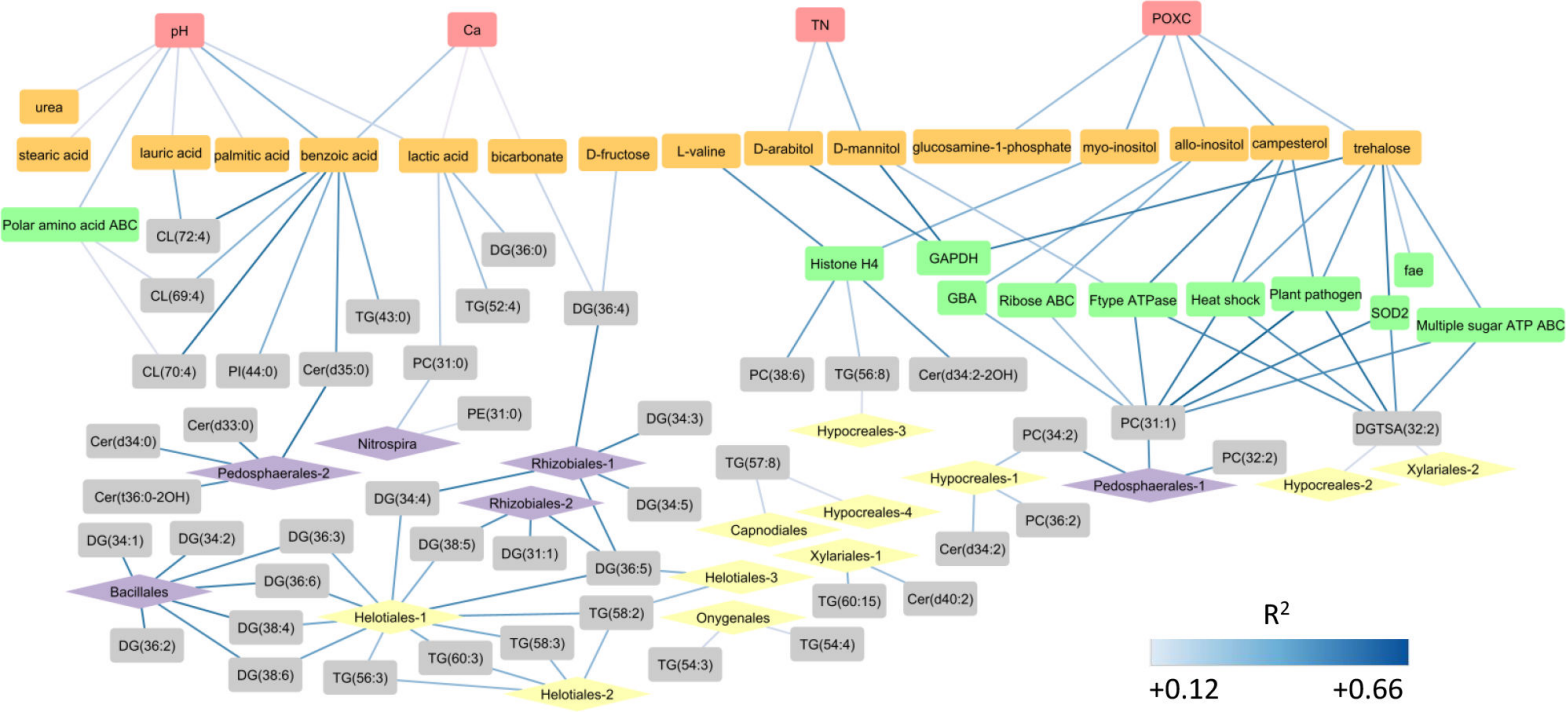
Strongest positive Spearman correlations (0.12 < *R* ^2^ < 0.66) between abiotic (absolute data) and log2 normalized multi-omics properties of interest averaged across all field treatments and soil depth increments. Abiotic soil properties are denoted by pink boxes; polar metabolites are denoted by orange boxes; proteins are denoted by green boxes; lipids are denoted by gray boxes; and amplicon sequence variants (ASVs) representing specific orders of bacteria and fungi are purple and yellow diamonds, respectively, and are labeled as their larger representative classes.

The polar metabolome composition was significantly impacted by both soil depth and field treatments (Fig. S1). The soil metabolome reflected abiotic gradients with soil depth as evidenced by significantly different metabolome compositions between the surface (0–5 cm) and deep (48–100 cm) soil horizons (non-metric multi-dimensional scaling [NMDS] permutational multivariate analysis of variance [PERMANOVA] *P*_adj_ < 0.01) (Fig. 2). Variations in the metabolomes were largely explained by soil pH (*R* ^2^ = 0.25, *P*_adj_ = 0.04) and POXC (*R* ^2^ = 0.21, *P*_adj_ = 0.04) in opposing directions along NMDS1 (Fig. 2; Table 2). Along with higher soil pH, the deep soil horizon contained significantly (*P*_adj_ < 0.05) higher levels (log2 normalized relative abundances) of bicarbonate and organic acids including benzoic acid, lactic acid, stearic acid, palmitic acid, and myristic acid (Fig. 1b; Table 1; Tables S2 and S4). There were distinct (Fig. 3A) and often significant (*P* < 0.01) positive Spearman correlations (Fig. 4) between abiotic and metabolomic soil properties of interest across all treatments and depths. For example, several of the organic acids (benzoic, lactic, palmitic, and stearic) were significantly correlated with soil pH, and bicarbonate was correlated with Ca concentrations (Fig. 4). Bicarbonate may be exuded by plant roots to lower the surrounding soil pH (39). Organic acids may also be microbially derived to dissolve CaCO_3_ minerals in deep calcareous soil horizons; previous studies have illustrated CaCO_3_^10^ and mineral (39) dissolution with organic acids. Soil pH and Ca concentrations are drivers of soil microbial CaCO_3_ precipitation (38), and positively correlated with the polar metabolites that were more abundant in deep soils as compared to surface soils (Fig. 3a). These findings suggest that polar metabolites may be involved in processes that influence CaCO_3_ precipitation.

**TABLE 2:** Correlations between abiotic soil properties and metabolomic, lipidomic, proteomic, and taxonomic ordinations^*b*^

Parameter	Metabolome		Lipidome		Proteome		16S amplicons		ITS amplicons^*a*^	
	*R* ^2^	*P*_adj_	*R* ^2^	*P*_adj_	*R* ^2^	*P*_adj_	*R* ^2^	*P*_adj_	*R* ^2^	*P*_adj_
TOC	*0.16*	*0.06*	0.57	0.00	0.30	0.01	0.29	0.01	*0.07*	*0.33*
TN	*0.08*	*0.44*	0.59	0.00	0.41	0.01	0.30	0.01	*0.08*	*0.33*
POXC	0.21	0.04	0.40	0.00	0.46	0.01	*0.10*	*0.19*	*0.10*	*0.30*
pH	0.25	0.04	0.51	0.00	0.33	0.01	*0.11*	*0.19*	0.32	0.02
SIC	*0.03*	*0.71*	*0.02*	*0.60*	*0.12*	*0.10*	*0.05*	*0.40*	*0.09*	*0.31*
Moisture	*0.01*	*0.84*	*0.13*	*0.06*	*0.00*	*0.98*	*0.01*	*0.74*	0.20	0.03
SOM	*0.05*	*0.52*	0.53	0.00	0.19	0.04	*0.10*	*0.19*	*0.02*	*0.80*
P	*0.07*	*0.45*	0.35	0.00	0.23	0.01	*0.07*	*0.33*	*0.07*	*0.33*
Ca	*0.01*	*0.84*	0.22	0.00	0.20	0.01	*0.03*	*0.60*	*0.06*	*0.41*
Na	*0.07*	*0.44*	*0.09*	*0.13*	*0.03*	*0.58*	*0.06*	*0.36*	0.40	0.01
Zn	*0.04*	*0.54*	0.38	0.00	0.21	0.02	*0.15*	*0.14*	*0.10*	*0.30*
Mn	*0.05*	*0.52*	0.48	0.00	0.31	0.01	*0.17*	*0.08*	*0.05*	*0.43*
Fe	*0.01*	*0.84*	0.26	0.00	0.27	0.01	*0.09*	*0.19*	*0.01*	*0.87*

^*a*^ ITS, intervening sequence.

^*b*^ Non-significant correlations are in italics.

In contrast to polar metabolites that were more abundant in the deep soil horizon, the metabolic signatures of surface soil horizons reflected plant and microbial responses to osmotic stress from greater fluctuations in soil moisture and temperature. The surface soil horizon had significantly (*P*_adj_ < 0.05) higher levels of low-molecular-weight sugars, sugar alcohols, and several other easily metabolized substrates (Fig. 1b; Table 1; Tables S2 and S4) compared to the deep soil horizon. Trehalose and sugar alcohols are produced by plants and microbes to regulate cellular osmotic potential under water, heat, and salt stress (40). Levels of sugars, such as trehalose and glucosamine-1-phosphate, and sugar alcohols, such as campesterol, myo-inositol, and allo-inositol were significantly (*P* < 0.01) positively correlated most with POXC concentrations (Fig. 3a and 4). The POXC method measures redox reactive SOC compounds, especially simple substrates with alcohol groups that are easily oxidizable (34). These results reveal the potential for chemical oxidation of soil C is highest at the soil surface where there are more microbial interactions with fluctuations in soil moisture.

Irrigation effects on the polar metabolome composition were less pronounced than depth effects. However, there were signals of osmotic stress responses to irrigation-supplied soil moisture. Unirrigated bare soils had significantly (*P*_adj_ < 0.05) higher levels of trehalose compared to irrigated bare soils (Fig. 1b; Table 1; Tables S2 and S4). Trehalose is a non-reducing disaccharide of D-glucose that serves as an osmoprotectant (40). Irrigated bare soils also had significantly (*P*_adj_ < 0.05) lower levels of D-mannose, an epimer of D-glucose and a precursor to trehalose, compared to unirrigated bare soils (Fig. 1b; Table 1; Tables S1b and S2). One possible explanation for these results is that under high osmotic stress in bare soils without irrigation, D-mannose may have been converted into D-glucose, which may have been further reduced via microbial metabolism into trehalose. The negative correlations of trehalose with soil moisture (Fig. 3) are consistent across soil depths and treatments. Our study is one of the first to show that trehalose can concurrently indicate different levels of osmotic stress with soil depth and across field treatments. Irrigation of bare soils also influenced the abundance of bicarbonate, urea, and L-valine—the latter two serve as sources of both C and N. The irrigated bare soils had significantly (*P*_adj_ < 0.05) lower bicarbonate and urea levels compared to unirrigated bare soils (Fig. 1b; Table 1; Tables S2 and S4). Urea was significantly positively correlated with soil pH (*P* < 0.01) (Fig. 3a and 4), which could be associated with urea hydrolysis that produces ammonia (NH_3_) and CO_2_, with CO_2_ further reacting with water to produce bicarbonate. However, the decrease in both urea and bicarbonate with irrigation suggests that the bicarbonate is leaching (9), or being transformed and reacting with exchangeable soil Ca in CaCO_3_ precipitation (38). These results suggest that the microbiome can influence specific biochemical interactions and drive transformations between organic and inorganic forms of soil C in associations with minerals. Another example is L-valine, a polar amino acid that responded differently to soil depth with and without irrigation (Fig. 1b). In unirrigated bare soils the surface soil horizon had lower levels of L-valine than the deep soil horizon. By contrast, when averaged across unplanted and planted irrigated soils, the surface soil horizon contained higher levels of L-valine. These findings exemplify how irrigation-controlled soil moisture impacts the solubility of specific metabolites and their accessibility to the microbiome.

The effects of tall wheatgrass on the soil metabolome composition were less pronounced than depth and irrigation (Fig. S1). However, soils planted with both varieties of tall wheatgrass had significantly (*P*_adj_ < 0.05) higher levels of bicarbonate, D-fructose, and tagatose compared to irrigated bare soils (Fig. 1b; Table 1; Tables S2 and S4). Bicarbonate ions can be exuded by roots and are generated when water reacts with CO_2_, suggesting that increased microbial respiration in the presence of sugars exuded from roots may contribute to enhanced bicarbonate production. This hypothesis is supported by significant positive correlations of bicarbonate with D-fructose (*R* = 0.36), a dominant sugar in root exudates (41) and tagatose (*R* = 0.37), a less common sugar (both *P* = 0.01). Together these findings suggest that water stimulates root exudation of simple sugars, which in turn stimulates microbial activity resulting in enhanced bicarbonate production. By contrast, in the absence of plants, the irrigated bare soils had the lowest levels of bicarbonate. Therefore, planting wheatgrass allowed for retention of inorganic C that was otherwise lost through irrigation. In addition, deep-rooted perennial grasses may recruit microbes capable of CaCO_3_ precipitation (38) under high soil pH and Ca^+2^ concentrations in calcareous soils. Under high Ca conditions, the microbiome was previously shown to precipitate soluble C and sequester it in soil under alfalfa and soybean (12). Our results therefore suggest that there is potential for microbial CaCO_3_ precipitation in calcareous soil horizons that is enhanced in the presence of deep-rooting perennial grasses and their associated microbiomes.

Plant root presence also impacted microbial metabolism of some organic and fatty acids. For example, benzoic, nonanoic, and lauric acids were significantly (*P*_adj_ < 0.05) higher in the absence of plant roots (Fig. 1b; Table 1; Tables S2 and S4). Like benzoic acid, lauric and nonanoic acids correlated with soil pH (Fig. 3a and 4). Soil planted with Alkar, but not Jose, had significantly (*P*_adj_ < 0.05) lower levels of capric acid than irrigated bare soils (Fig. 1b; Table 1; Tables S2 and S4), suggesting that the microbial metabolism of certain fatty acids differs between cultivars. Depletion of fatty acids in the soil may be related to the presence of arbuscular mycorrhizal fungi, which are fatty acid auxotrophs (42). The lower levels of nonanoic, lauric, and capric acids suggest that they were metabolized or transformed by plant-associated microbes as saturated fatty acids that can inhibit the mycelial growth of phytopathogenic fungi (43).

### Soil lipidome reveals significant differences with soil depth that outweigh field treatment effects

The soil lipidome was the most discriminatory of the omics data sets. The untargeted soil lipidomics approach that we employed reflected many sources of lipid C compounds that could be potentially stored or alternatively lost through decomposition, including those that are mineral-associated and/or plant/microbially derived, as part of the SOC pool (23, 44). Our analysis identified 649 lipids including glycerophospholipids and non-phosphorus-containing glycerolipids and sphingolipids.

There were significant differences in the composition of lipids in surface and deep horizons (Fig. S1; Table S4). Triacylglycerols (TG) represented the largest lipid class and constituted 31% of the lipids, followed by 19% glycerophosphocholines (PC). These two lipid classes showed different responses to soil depth; several PC (*n* = 39) were more abundant at the soil surface in contrast to higher abundances of TG (*n* = 46) in deeper soils (Fig. S1; Table S4). Higher levels of PCs in surface soils likely reflect their roles as osmoprotectants for both plants and microorganisms (45, 46). The contrasting higher amounts of TG lipids in deep soils suggest that they accumulated as energy storage compounds by specific members of the soil microbiome or plant roots, at depth (47, 48).

The lipidome was sensitive to osmotic and oxidative stress in the soil profile. In general, lipids with 0 or 2 degrees of unsaturation tended to increase in abundance with depth, while those that decreased in abundance had higher degrees of unsaturation. Specifically, saturated lipids comprised 50% (*n* = 34) of the 68 lipids that increased in abundance and 18% (*n* = 14) of the 77 lipids that decreased in abundance with depth (Table S4). The saturated lipids in deeper soils may be derived from microbial membranes, as saturation increases structural stability and decreases susceptibility to oxidation (49). On the other hand, the higher amount of unsaturated lipids at the soil surface may be associated with a higher abundance of plant roots and fungi that are known to contain more unsaturated lipids compared to those from bacteria or archaea (47). Higher degrees of unsaturation in plant membrane lipids have also been shown to improve tolerance to salt stress (50) and may have also been produced as a stress response to the surface soil conditions.

**FIG 5:**
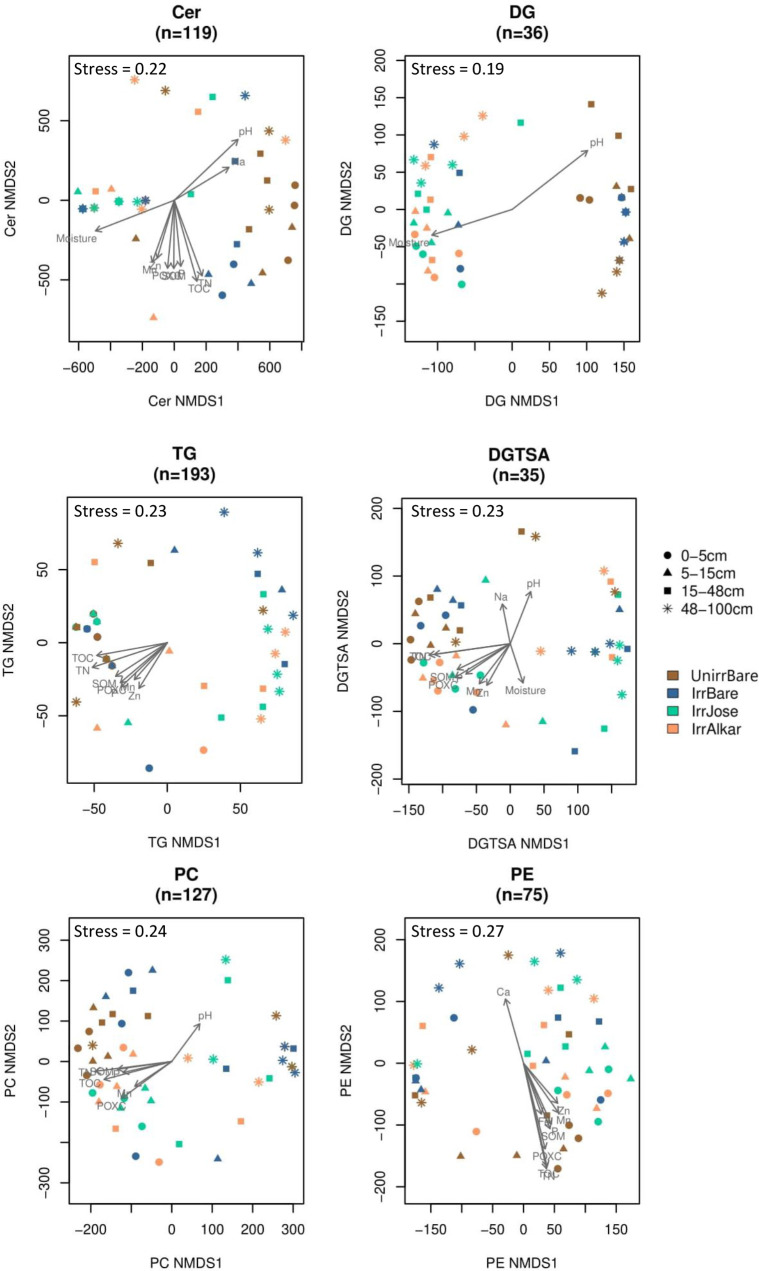
Lipidomic differences with soil depth and treatment. Dissimilarities were visualized using NMDS and were tested for correlations with abiotic soil properties and differences with soil depth and field treatment. A single core was sampled from three replicate blocks of four treatments (i.e., unirrigated bare [brown], irrigated bare soil [blue], irrigated Alkar [orange], and irrigated Jose [green]) and broken into four depth increments from 0 to 5 cm (circles), 5–15 cm (triangles), 15–48 cm (squares), and 48–100 cm (stars) to produce 48 soil samples.

Ceramides responded to irrigation-controlled soil moisture and provided details about phenotypic adaptations to the irrigation treatment. Three saturated ceramides were significantly lower in irrigated bare soils compared to unirrigated bare soils, including two hydroxylated ceramides (Table S4). Hydroxylated ceramides enable eukaryotic cellular membrane homeostasis and signaling (51) and are regulators of osmotic stress in eukaryotes (52) and some bacteria (53). Variations in ceramides were explained by differences in soil moisture (*R* ^2^ = 0.31), in addition to soil pH (*R* ^2^ = 0.34), TOC (*R* ^2^ = 0.31), and TN (*R* ^2^ = 0.28) (all *P*_adj_ < 0.01) (Fig. 5; Table 3). Select ceramides were correlated to fungal ASVs (*Ascomycota* and *Basidiomycota*) and bacterial ASVs (*Verrucomicrobiota*) (Fig. 4). *Verrucomicrobiota* which are common soil bacteria with few cultured representatives, however, members of this phyla are recognized for their ability to live in low resource environments and degrade complex C compounds in prairie soils (54). It is noteworthy that the sequenced *Verrucomicrobium* sp. IMCC26134 has a pathway for sphingolipid metabolism (KEGG IMCC26134_9670) (55). Our results suggest that members of the *Ascomycota, Basidiomycota,* and *Verrucomicrobia* phyla respond to decreases in soil moisture by regulating ceramides in their cell membranes. Alternatively, these specific microbial populations are enriched under low moisture conditions and the ceramides reflect the biomass of the cells. We note in this example, and elsewhere, that it is difficult to determine whether a change in abundance of a specific lipid is due to a change in cell abundance where the lipids remain static, or due to changes in select lipids due to physiological adaptations to environmental conditions, where the cell number remains static.

**TABLE 3:** Correlations between abiotic soil properties and class-level lipidomic ordinations^*a*^

Parameters	Ceramide		Diacylglycerol		Triacylglycerol		Betaine		Phosphocholine		Phosphoethanolamine	
	*R* ^2^	*P*_adj_	*R* ^2^	*P*_adj_	*R* ^2^	*P*_adj_	*R* ^2^	*P*_adj_	*R* ^2^	*P*_adj_	*R* ^2^	*P*_adj_
TOC	0.31	0.00	*0.18*	*0.07*	0.35	0.01	0.51	0.00	0.46	0.00	0.53	0.00
TN	0.28	0.00	*0.12*	*0.16*	0.42	0.01	0.59	0.00	0.57	0.00	0.56	0.00
POXC	0.20	0.02	*0.10*	*0.17*	0.26	0.01	0.37	0.00	0.37	0.00	0.37	0.00
pH	0.34	0.00	0.32	0.01	*0.09*	*0.16*	0.27	0.00	0.21	0.02	*0.09*	*0.14*
SIC	*0.01*	*0.89*	*0.10*	*0.17*	*0.04*	*0.42*	*0.02*	*0.55*	*0.02*	*0.69*	*0.03*	*0.49*
Moisture	0.31	0.00	0.25	0.03	*0.07*	*0.24*	0.15	0.03	*0.09*	*0.14*	*0.13*	*0.07*
SOM	0.20	0.02	*0.07*	*0.28*	0.26	0.01	0.30	0.00	0.28	0.00	0.24	0.01
P	0.19	0.03	*0.04*	*0.45*	0.28	0.01	0.25	0.00	0.24	0.01	0.21	0.01
Ca	*0.04*	*0.45*	*0.08*	*0.24*	*0.08*	*0.19*	*0.13*	*0.06*	*0.10*	*0.14*	0.21	0.02
Na	0.18	0.03	*0.15*	*0.07*	*0.02*	*0.71*	0.14	0.04	*0.03*	*0.55*	*0.05*	*0.39*
Zn	0.16	0.03	*0.06*	*0.30*	0.20	0.01	0.20	0.01	*0.14*	*0.06*	0.13	0.05
Mn	0.19	0.03	*0.15*	*0.07*	0.17	0.04	0.22	0.01	0.19	0.02	0.18	0.03
Fe	*0.08*	*0.22*	*0.03*	*0.48*	*0.14*	*0.06*	*0.08*	*0.16*	*0.13*	*0.07*	0.14	0.05

^*a*^ Non-significant correlations are in italics.

Among all the -omics data, the lipidome composition was the most responsive to tall wheatgrass treatments (Fig. 2; Fig. S1). The lipidome response was most striking for the composition of diacylglycerides (DG) (Fig. S1; Fig. 5), which have been associated with drought stress in plants (56). Variation in DG lipids was correlated with soil pH (*R* ^2^ = 0.32; *P*_adj_ = 0.01) and soil moisture levels (*R* ^2^ = 0.25, *P*_adj_ = 0.03) (Fig. 5; Table 3), both of which are properties that were impacted by irrigation. Planted soils had significantly (*P*_adj_ < 0.05) higher abundance of several DG lipids with 16 and 18 C fatty acids and 3–5 double bonds compared to irrigated bare soils (Table S4). Analysis of the lipidome in soil is relatively new and still lacks taxonomic annotation. Although physiological adaptation may or may not be reflected in changes in taxonomic abundance, at present correlation between specific lipids and ASVs highlights the potential of lipids for determining the physiological status of target microbial populations in the soil (57). For example, in a prior study of grassland surface soil, glycerolipids containing 34 and 36 C and 2–6 degrees of unsaturation were attributed to plants and fungi (44). However, we found that DG lipids were most strongly correlated to ASVs corresponding to potential plant growth promoting bacteria (Fig. 4), including members of the Firmicutes (*Bacillales*) (58) and Proteobacteria (*Rhizobales*) (59); the latter includes some members capable of N fixation.

There were significant correlations between TG, PC, phosphoethanolamine (PE), and betaine (DGTSA) lipids and soil N. Variations in these lipid classes were better explained by TN than TOC (Fig. 5; Table 3), which suggests that microbial synthesis of these lipids is more governed by N availability than C. DGTSA lipids were the most strongly correlated lipid class to TN (*R* ^2^ = 0.59), followed by PC (*R* ^2^ = 0.57), PE (*R* ^2^ = 0.56), and TG (*R* ^2^ = 0.42) (Table 3). Deep soils contained significantly higher levels of select TG lipids containing N, and PE lipids containing N and P (Fig. S1; Table S4), but lower concentrations of several PC and DGTSA lipids. These differences could reflect physiological responses of soil microbes to low availability of N and P in deeper soil layers, as previously found in calcareous soils (60, 61), and in this study (Fig. 1a; Table S1). We hypothesize that the DGTSA lipids, which contain ammonium, are rapidly metabolized by the ammonia oxidizing bacteria (*MND1*) and archaea (*Candidatus Nitrosotenuis*) that were more abundant in deep soils based on sequencing data (Fig. S2; Table S5). Ammonia oxidation could be enhanced in deeper soil to replenish cellular N concentrations (62) and/or to produce nitrate, which would further reflect N limitation at the field site (Table S1a). By contrast, the higher abundance of DGTSA in the surface soils could be derived from fungi that synthesize DGTSA under P-limiting conditions (63). This hypothesis is strengthened by correlations between fungal Ascomycota ASVs and select TG, DG, and DGTSA lipids (Fig. 4). Fungi are known to synthesize TG under N limitation (64). For example, long chain glycerolipids with two or more degrees of unsaturation were correlated with several orders within the Ascomycota phylum, including *Helotiales, Hypocreales, Onygenales, Capnodiales,* and *Xylariales* (Fig. 4). In particular, 11 *Helotiales* ASVs were highly correlated with 12 TG, 8 DG, and 1 DGTSA (Fig. 4). The *Helotiales* order includes root-associated ectomycorrhizae that promote plant uptake of P under P-limited conditions (65). The Ascomycota phylum often predominates over other fungal phyla in low resource environments and under drought stress (66). Our results highlight the importance of considering interactions and bioavailability of different forms of N and P when studying soil C cycling.

### Soil proteomes are impacted more significantly by soil depth than irrigation or plants

Congruent with the polar metabolome and lipidome, the proteome composition differed more with soil depth than with irrigation or plants. Similar to other omics data, proteomic responses to osmotic stress were observed in the surface soil. The surface soil horizon (0–5 cm) had significantly (*P*_adj_ < 0.05) higher levels of superoxide dismutase in the Fe-Mn family (SOD2) compared to the deep soil horizon (48–100 cm) (Fig. 1c; Table 1; Tables S3 and S4). The SOD2 protein is produced as an oxidative stress response to reactive oxygen species (67). Also, SOD2 was significantly (*P* < 0.01) correlated with trehalose in the metabolite data (Fig. 4), indicative of physiological adaptations to stress conditions on the soil surface. The surface soil horizon also had significantly (all *P*_adj_ < 0.05) higher levels of glucosylceramidase (GBA), ribose ABC transporters, and multiple sugar ATP-binding ABC transporters (Fig. 1c; Table 1; Tables S3 and S4). The GBA and ribose ABC transporter proteins were significantly (*P* < 0.01) correlated with allo-inositol in the metabolite data, whereas the multiple sugar ATP-binding ABC transporter protein significantly correlated with trehalose (*P* = 0.01) (Fig. 4). The sugar ABC transporters could be involved in taking up sugars released from GBA and/or from the oxidation of sugar alcohols. GBA hydrolyzes glycosyl compounds to produce D-glucose and ceramide lipids (68). Elevated ceramide levels have been associated with experimentally induced drought stress in these soils (57). Interconversions of sugars and sugar alcohols may be facilitated by alcohol dehydrogenase (69) or glyceraldehdyde 3-phosphate dehydrogenase (GAPDH) (70), both of which are oxidoreductase enzymes involved in glycolysis. This hypothesis is supported by significant (*P* < 0.01) correlations of GAPDH with D-arabitol and D-mannitol metabolites (Fig. 4). Another protein that was significantly (*P*_adj_ < 0.05) higher in surface soils was a formaldehyde activating hydrolase enzyme (fae) (Fig. 1c; Table 1; Table S1) that is involved in the methane cycle (71), suggesting that methane or methanol was produced and/or consumed to a greater extent in surface soils.

By contrast to the proteins that were more abundant in the surface soil horizon, the deeper 15- to 48-cm and 48- to 100-cm soil horizons had significantly (*P*_adj_ < 0.05) higher levels of L-amino acid ABC transporters compared to the 0- to 5-cm and 5- to 15-cm soil horizons (Fig. 1c; Table 1; Tables S3 and S4). The 15- to 48-cm soil horizon also had higher levels of polar amino acid and alpha-glucoside ABC transporters compared to the 0- to 5-cm soil horizon (Fig. 1c; Table 1; Tables S3 and S4). The higher levels of these ABC transport proteins could reflect higher microbial consumption or production of amino acids and alpha-glucosides in the deeper soil horizons. In the planted treatments in particular, the 15- to 48-cm soil horizon could have been a hotspot for rhizodeposition of amino acids (18) which could have stimulated higher activity of polar and L-amino acid transport proteins.

Soil depth and plant treatments had effects on F-type H^+^/Na^+^ ATPases and histone H4 proteins. The surface soil horizon also had significantly (*P*_adj_ < 0.05) higher levels of F-type H^+^/Na^+^ ATPase (beta subunit) compared to the deep soil horizon (Fig. 1c; Table 1; Tables S3 and S4). The ATPase subunits facilitate plant photosynthesis and oxidative phosphorylation in bacteria (72). The alpha subunit of ATPase was also significantly more abundant under irrigated soils planted with Alkar compared to irrigated bare soils (Fig. 1c; Table 1; Tables S3 and S4), suggesting that they are plant derived and/or are associated with plant-associated bacteria. The ATPase alpha and beta subunit proteins were significantly correlated with campesterol (Fig. 4), which is the precursor of brassinosteroids (73), involved in numerous physiological plant processes including soil water balance and drought stress mitigation (74, 75). Additionally, histone H4 was significantly more abundant in surface soils and the planted treatments (Fig. 1c; Table 1; Tables S3 and S4) and was significantly correlated with myo-inositol (Fig. 4). Histone proteins could be plant derived (76), or from plant-associated fungi (64). However, it is currently difficult to discern whether these proteins and metabolic products are derived from plants or fungi because they are highly conserved in eukaryotes.

Like the metabolome and lipidome, variations in the soil proteome (using the top 50 identified proteins listed in Table S6) were best explained by levels of POXC (*R* ^2^ = 0.46) and soil pH (*R* ^2^ = 0.33) (both *P*_adj_ = 0.01) in opposing directions along NMDS1 (Fig. 2; Table 2). The strong correlations of POXC with the multi-omics analyses in our study align with the previous study that found that POXC was a strong predictor of differences in bacterial metagenomes (36). Also, similar to the soil lipidome, variations in the soil proteome were correlated with concentrations of TN (*R* ^2^ = 0.41), Mn (*R* ^2^ = 0.31), and Fe (*R* ^2^ = 0.27) (all *P*_adj_ = 0.01) (Fig. 2; Table 2). Strong correlations of Fe and Mn with the proteome and lipidome highlight soil micronutrient impacts on protein and lipid metabolism. Fe- and Mn-containing minerals are cofactors in extracellular enzyme activities and as oxides in microbial redox reactions that influence SOM decomposition (77). Thus, differences in soil chemistry may impact microbial metabolism of C that is reflected in metabolites, lipids, and proteins in surface and deep soils. In general, the protein data complemented the metabolite and lipid data in providing a more detailed view of plant and microbial physiological responses to soil depth and treatment.

### Summary

This study provides a deep multi-omics analysis of plant and microbial interactions that are potential drivers of organic and inorganic soil carbon pools in an irrigated and unfertilized calcareous soil. The resulting data reveal how the combined phenotypes of the soil microbiome, i.e., the metaphenome, varied with soil depth in the calcareous soils. Specifically, the metabolomics, lipidomics, and proteomics data revealed several potential signatures of nutrient or moisture stress that varied with soil depth and field treatments. A potential driver of soil microbial CaCO_3_ precipitation in deep calcareous soils was the increase in soil pH with depth. In turn, pH was strongly correlated with polar metabolites, lipids, and proteins. In addition, Ca concentrations were strongly correlated with lipids and proteins. These results align with previous research that shows that abiotic soil properties drive CaCO_3_ formation and dissolution in calcareous soils. Here we show that these changes also impact soil organic carbon accumulation and soil organic matter decomposition by soil microorganisms.

Soil carbon chemistry is normally limited to measuring bulk categories of compounds without knowledge of which specific compounds are included in the analyses. Here, we found that measurement of oxidizable organic carbon compounds was correlated to the abundances of specific sugars and sugar alcohols that are easily oxidizable and related sugar transport proteins (Fig. 4). The accumulation of these C compounds in the surface soil is likely a general product of an osmotic stress response of the microbiome because they were observed under all field treatments, including in the absence of plants. This is the first demonstration of significant correlations between specific sugars, sugar alcohols, and proteins involved in sugar uptake and metabolism with POXC, a traditional chemical assay of oxidizable soil carbon compounds, using soils that are not manipulated in the lab. This finding has helped to illuminate how specific soil carbon compounds and cycling processes underlie traditional POXC measurements. Together, the POXC and omics data reveal that simple carbon compounds are cycled in surface soils under osmotic and oxidative stress under low soil moisture conditions.

Throughout the soil profile and across different treatments, differences in soil pH and concentrations of POXC, N, and Ca strongly correlated with differences in multi-omics data. To our knowledge, this is the first study to make such a detailed comparison of multi-omics and soil chemical field data. Specifically, we found that deep-rooted perennial grasses promoted retention of bicarbonate that decreased with irrigation. However, although levels of SOC and SIC decreased with irrigation, they did not significantly increase with plant cover. Several microbial taxa in the deep soil were positively correlated to soil pH and Ca^+2^ levels, suggesting they were adapted to these conditions and may participate in CaCO_3_ dissolution or precipitation in calcareous soils. This study highlights the importance of considering microbial interactions with organic, inorganic, and oxidizable soil carbon fractions in unfertilized, marginal soils with calcareous soil horizons as potential resources for retaining soil organic carbon as the climate changes. Lastly, this study is novel in providing examples of specific compounds that reflect differences in carbon chemistry with and without irrigation and plant cover at different soil depths.

## MATERIALS AND METHODS

### Field experimental design and segmented deep soil sampling

A randomized and replicated field trial was established in 2018 at the Washington State University IAREC in Prosser, WA (46°15′04″N and 119°43′43″W). This previously uncultivated site is in an arid location with a mean annual precipitation of 160 mm and a mean daily summer (June–August) temperature range of 55 to 87°F (78). The marginal soil is classified as a Warden silt loam that is characterized as a coarse-silty, mixed, superactive, mesic Xeric Haplocambid (79) with low organic matter and alkaline soil pH. Prior to planting two varieties of perennial tall wheatgrass (*Thinopyrum ponticum*), the field was burned and treated with herbicide 2,4-dichlorophenoxyacetic acid (2,4-D) to remove native plant cover. Three replicate blocks of four treatments were sampled, including unirrigated bare soils, irrigated bare soils, and irrigated soils cultivated with tall wheatgrass varieties “Alkar,” a northern ecotype bred for the Pacific Northwest (80), and “Jose,” a southern ecotype bred in New Mexico (31). In the irrigated treatments, surface drip irrigation was applied weekly based on crop evapotranspiration monitored by Washington State University at a rate of 18.75% of the recommended application to impose drought stress.

A week following aboveground biomass harvest on 20–21 July 2020, one soil core was extracted from triplicate blocks for each of the four treatments with a tractor mounted hydraulic Kauffman probe. Each soil core (1 1/4 inch in diameter) was sampled from an individual plot from 0- to 100-cm depth. Then each soil core was split at depth increments (horizons) of 0–5 cm (Ap1), 5–15 cm (Ap2), 15–48 cm (AB), and 48–100 cm (2Bk) to distinguish topsoil effects and to match diagnostic horizons of the Warden soil series. Each horizon sample (*N* = 48) was homogenized individually in a sterilized, labeled foil packet with some root material, if present, to represent the bulk soil. Approximately 30 g of each sample was subsampled for abiotic soil properties, and the remaining sample was stored at −80°C for biotic soil properties.

### Abiotic soil properties

Soil moisture as gravimetric water content was measured by weight loss upon heating and drying frozen samples at 105°C for 72 hours. Soil pH of standard 1:1 soil:H_2_O solution was measured using a calibrated pH meter and electrode (Mettler-Toledo, Columbus, OH, USA). Total soil organic carbon (TOC) and inorganic carbon (SIC) and total nitrogen (TN) were measured by rapid combustion using a LECO TruSpec (St. Joseph, MI, USA) at the WSU Soil and Plant Analysis Lab in Pullman, WA, USA. TOC and SIC were differentiated using acid pretreatment to remove inorganic C (thought to be calcium carbonates [CaCO_3_]) from a secondary set of samples (81). Permanganate oxidizable carbon was measured using potassium permanganate to oxidize SOC and to measure the reduction in bleaching solution spectrophotometrically (82 – 84). Briefly, 5-g air-dried soil was added to 50-mL Falcon tubes with 18 mL of deionized water and 2 mL of 0.2 M KMnO_4_. The tubes were shaken on an oscillating shaker for 2 min at 240 oscillations/min and then allowed to settle for 10 min at room temperature in the dark. The solution was diluted 1:100 with deionized water, then 250 μL of each sample was loaded into the column of a 96-well plate containing a column of standards and blanks. Sample absorbance was read at 550 nm with a spectrophotometric plate reader (BioTek Instruments, Winooski, VT, USA). Additional soil chemical analyses were completed at Kuo Testing Laboratory (Othello, WA, USA), and the procedures used were the Walkley-Black method for SOM (85); Olsen (NaHCO_3_) extractant for phosphorus (37); NH_4_OAc (pH 8.5) extractant modified for exchangeable base cations (i.e., K^+^, Ca^+2^, Mg^+2^, and Na^+^) (86); and DTPA extractant for S and metal micronutrients (i.e., Mn^+2^, Zn^+2^, and Fe^+2^) (87).

### Amplicon sequence variants

Using soil samples stored at −80°C, total DNA was extracted from 250 mg of soil using a Zymo Quick-DNA Fecal/Soil Microbe Miniprep Kit according to manufacturer instructions with optimization using β-mercaptoethanol (ZymoResearch, Irvine, CA, USA). Sequencing was performed by Argonne National Laboratory on an Illumina MiSeq instrument (Illumina, San Diego, CA, USA). Triplicate, separate 16S and intervening sequence (ITS) rRNA gene amplification reactions were performed by targeting the prokaryotic 16S V4/V5 region with the primer pair 515F (5ʹ GTGCCAGCMGCCGCGGTAA 3ʹ) and 806R (5ʹ GGACTACHVGGGTWTCTAAT 3ʹ) by Illumina MiSeq 2 × 151 bp paired-end sequencing, and the fungal ITS-1 region with the primer pair ITS1f (5ʹ CTTGGTCATTTAGAGGAAGTAA 3ʹ) and ITS2 (5ʹ GCTGCGTTCTTCATCGATGC 3ʹ) by Illumina MiSeq 2 × 250 bp paired-end sequencing, according to the Earth Microbiome protocols for 16S and ITS, except for the use of forward barcoded 16S primers (88). QIIME2 (89) was used to process the high-throughput, paired-end 16S and ITS signature amplicon sequence reads and to generate amplicon sequence variants (ASVs). 16S and ITS ASV taxonomies were assigned using the SILVA database (90) and the UNITE database (91), respectively.

A total of 15,460 bacterial, 301 archaeal, and 2,556 fungal amplicon sequence variants were identified.

### Metabolite, protein, and lipid extraction from soil

Soil was weighed into 50-mL methanol/chloroform compatible tubes (Genesee Scientific, San Diego, CA, USA) along with 10 mL of 0.9- to 2.0-mm stainless steel beads, 0.1-mm zirconia beads and 0.1-mm garnet beads (Next Advance, Troy, NY, USA). All beads had previously been washed with chloroform and methanol and dried in a fume hood. Metabolites, proteins, and lipids were extracted using a modified Folch extraction (92) specifically for soil as previously described (33).

### Lipidomics analysis

A Waters NanoAquity UP Liquid Chromatography (LC) system interfaced with a Velos-ETD Orbitrap mass spectrometer (Thermo Scientific, San Jose, CA, USA) was used for liquid chromatography-electrospray ionization-mass spectrometry (LC-ESI-MS/MS) analyses. Total lipid extracts were dried and reconstituted in 100% methanol and injected onto a Waters column (HSS T3 1.0 mm × 150 mm × 1.8 µm particle size). Lipids were separated over a 90-min gradient elution (mobile phase A: ACN/H2O [40:60] containing 10 mM ammonium acetate; mobile phase B: ACN/IPA [10:90] containing 10 mM ammonium acetate) at a flow rate of 30 µL/min. Samples were analyzed in both positive and negative ionizations using higher-energy collision dissociation (HCD) and collision-induced dissociation (CID) to obtain high coverage of the lipidome. LC-MS/MS raw data files were imported into the in-house developed software Lipid Informed Quantitation and Identification (LIQUID) for semi-automated identification of lipid molecular species. Confident lipid identifications were determined by examining the tandem mass spectra for diagnostic ion fragments along with associated chain fragment information. In addition, the isotopic profile, extracted ion chromatogram, and mass error of measured precursor ion were examined for lipid species. To align and gap-fill the mass spectrometry data, the identified lipid name, observed *m*/*z*, and the retention time from each analysis were used as a target database for feature identification across all liquid chromotography-mass spectrometry (LC-MS/MS) runs. This was achieved by aligning all data sets (grouped by ionization type) and matching unidentified features to their identified counterparts using MZmine version 2 (93). Aligned features were manually verified, and peak apex intensity values were exported for statistical analysis (94).

A total of 649 lipids were identified using liquid chromotography-mass spectrometry (LC-MS), including glycerophospholipids (129 glycerophosphocholines, 77 glycerophosphoethanolamines, 35 glycerophosphoglycerols, 9 glycerophosphoinositols, and 5 cardiolipins) and non-phosphorus-containing lipids (glycerolipids, including 204 triacylglycerols, 42 diacylglycerols, and 25 betaine lipids; and sphingolipids, including 120 ceramides).

### Metabolite derivatization and GC analysis

Metabolites were derived following previous published methods (95). The retention indices were first calculated based on the analysis of a mixture of fatty acid methyl esters (FAMEs), then this retention indexing information was subsequently applied to data from each sample to enable proper chromatographic alignment of each data set. Gas chromotography-mass spectrometry (GC-MS) raw data file processing was done using Metabolite Detector (96) software, and metabolites were identified by matching experimental spectra and retention indices to an augmented version of FiehnLib (97). All identifications were manually validated to reduce deconvolution errors and to eliminate false identifications. The NIST 14 GC-MS library was also used to cross-validate the spectral matching scores obtained using the Agilent library and to provide identifications of unmatched metabolites.

A total of 336 polar metabolite features were detected using gas chromotography-mass spectrometry (GC-MS), including 60 known polar including 60 known polar metabolites: 12 alcohols, 11 organic acids, 10 monosaccharides, 7 polysaccharides, 7 amino acids, 8 fatty acids, bicarbonate, and 4 N-containing compounds.

### LC-MS/MS proteomic analysis

A Waters nano-Acquity dual pumping UPLC system (Milford, MA, USA) was configured for online trapping of a 5-µL injection at 5 µL/min for 10 min followed by reverse-flow elution onto the analytical column at 200 nL/min. The trapping column was slurry packed in-house using 360 µm o.d. × 150 μm id fused silica (Polymicro Technologies Inc., Phoenix, AZ, USA) Jupiter 5-µm C18 media (Phenomenex, Torrence, CA, USA) with 2-mm sol-gel frits on either end. The analytical column was slurry packed in-house using Waters BEH 1.7-µm particles packed into a 35-cm-long, 360 µm o.d. × 75 μm i.d. column with an integrated emitter (New Objective, Inc., Littleton, MA, USA). Mobile phases consisted of 0.1% formic acid in water (mobile phase A) and 0.1% formic acid in acetonitrile (mobile phase B) with the following gradient profile (min, % B): 0, 1; 10, 8; 105, 25; 115, 35; 120, 75; 123, 95; 129, 95; 130, 50; 132, 95; 138, 95; and 140, 1.

MS analysis was performed using a Thermo Eclipse mass spectrometer (Thermo Scientific). The ion transfer tube temperature and spray voltage were 300°C and 2.4 kV, respectively. Data were collected for 120 min following a 27-min delay from when the trapping column was switched inline with the analytical column. Field asymmetric ion mobility spectrometry (FAIMS) was used at compensation voltage (CV) −40 V, −60 V, and −80 V. FT-MS spectra were acquired from 300 to 1800 *m*/*z* at a resolution of 120 k (AGC target 4e5) and while the top 12 FT-HCD-MS/MS spectra were acquired in data-dependent mode with an isolation window of 0.7 *m/*z and at an orbitrap resolution of 50 k (AGC target 5e4) using a fixed collision energy (HCD) of 32 and a 30-s exclusion time.

### Proteomic data analysis

The LC-MS/MS spectra were searched using the MS-GF+ search engine (98) against metaproteome database WA_2.5Tco_20210120.contig_2500.nr_1.0_ TrypPigBov_2021–07-21.fasta (15 + M protein sequences). The spectrum level peptide confidence score of the peptide-spectrum match (i.e., MSGFDB_SpecEValue) and mass difference (in ppm) between the observed parent ion and the computed mass of the identified peptide (i.e., DelM_PPM in MS-GF+) values were optimized to achieve the highest number of peptide identification within each data set while keeping the target decoy-based false discovery rate (FDR) of peptide identification below 5%.

A total of 19,165 proteins were detected using untargeted, shotgun proteomics LC-MS analysis. These proteins were dereplicated down to 1,623 known proteins with assigned Kyoto Encyclopedia of Genes and Genomes (KEGG) Orthology (KO) identification numbers. The 50 most common proteins, which were identified in at least 38 out of 48 soil samples, included 19 transport proteins and 17 enzymes.

### Statistical analyses

All statistical analyses were completed using R (version 4.0.2) (99) and functions in packages “vegan” (100) and “phyloseq” (101) unless otherwise noted. All graphs were made using the “ggplot2” package (102). It should be noted that all 48 soil samples were biological replicate samples from unique plot/depth combinations (four treatments × three blocks × four depths). Soil metabolomes, lipidomes, and proteomes were normalized using log2 transformation and central (median) tendency normalization. Soil samples were analyzed for soil microbial community structures based on 16S rRNA gene sequences for prokaryotes and intervening sequences for eukaryotes. Unique gene sequences were assigned as amplicon sequence variants. Prior to distance and dissimilarity measurements, ASVs were normalized using cumulative sum scaling with the “metagenomeseq” package (103).

Soil samples were analyzed for differences in taxonomy, metabolome, and lipidome composition over experimental variables of depth, irrigation, and plants using log2 fold changes. Log2 fold changes for 16S and ITS ASVs were calculated by differential gene expression analysis in the “DESeq” package (104) in the identical groupings as described below for multi-omics. Soil multi-omics fold changes with depth were constructed using differences in average normalized abundances in the top 0–5 cm (*N* = 12) and 48–100 cm (*N* = 12) soil horizons. Multi-omics fold changes with irrigation were calculated using differences in average log2 normalized abundances in the unirrigated (*N* = 12) and irrigated (*N* = 12) bare soils. Multi-omics fold changes with plant cultivar were calculated using differences in average normalized abundances in the irrigated bare soil (*N* = 12) with irrigated Alkar (*N* = 12) and Jose (*N* = 12). Additional pairwise comparisons of all four sampled horizons (comparing 0–5, 5–15, 15–48, and 48–100 cm) and combined irrigation/plant treatments were tested among sample replicates using the “aov()” function. Abiotic soil properties were also tested for differences among soil horizons and irrigation/plant treatments using the aov() function. There were no significant interaction effects on any of the abiotic or biotic soil properties, so only main effects are reported in this article. All *P*_adj_ values reported were adjusted for multiple group comparisons using the “TukeyHSD()” function and were calculated using the false discovery rate method.

Bray-Curtis distance matrices of multi-omics data were used to measure homogeneity of dispersion between experimental groups of treatment and depth. Dissimilarities in microbiome structure were visualized using non-metric multi-dimensional scaling. Pairwise comparisons were performed on group factors fitted to each NMDS using the “pairwise.factorfit()” function in “RVAideMemoire” package (105). Correlations of soil chemical properties and multi-omics in NMDS were tested using the “envfit() function,” and *P* values were adjusted with the FDR method using the “p.adjust.envfit()” function (106). Spearman correlations of key multi-omics and abiotic soil properties across all samples were calculated using the “cor()” function and were visualized as a network plot in Cytoscape (107).

A total of 649 lipids were identified using liquid chromotography-mass spectrometry (LC-MS), including glycerophospholipids (129 glycerophosphocholines, 77 glycerophosphoethanolamines, 35 glycerophosphoglycerols, 9 glycerophosphoinositols, and 5 cardiolipins) and non-phosphorus-containing lipids (glycerolipids, including 204 triacylglycerols, 42 diacylglycerols, and 25 betaine lipids; and sphingolipids, including 120 ceramides).

## Data Availability

The metadata and experimental data can be accessed here: https://data.pnnl.gov/group/nodes/dataset/33509. The raw sequencing data can be accessed here: https://data.pnnl.gov/group/nodes/dataset/33508.

## SUPPLEMENTAL MATERIAL

Figure S1 mbio.01758-23-s0001.pdf Changes in metabolites.

Figure S2 mbio.01758-23-s0002.pdf Shifts in taxa.

Figure S3 mbio.01758-23-s0003.pdf Relative taxon abundance.

Table S1 mbio.01758-23-s0004.pdf Abiotic soil properties.

Table S2 mbio.01758-23-s0005.pdf Polar metabolites.

Table S3 mbio.01758-23-s0006.pdf Protein abundances.

Table S4 mbio.01758-23-s0007.pdf Significant differences in metabolites, proteins and lipids.

Table S5 mbio.01758-23-s0008.pdf Differential gene abundances.

Table S6 mbio.01758-23-s0009.pdf Common proteins.

ASM does not own the copyrights to Supplemental Material that may be linked to, or accessed through, an article. The authors have granted ASM a non-exclusive, world-wide license to publish the Supplemental Material files. Please contact the corresponding author directly for reuse.
