# Supplementary material for: Influence of soil depth, irrigation, and plant genotype on the soil microbiome, metaphenome, and carbon chemistry
Source: mBio. 2023 Sep 20;14(5):e01758-23. doi: 10.1128/mbio.01758-23 (PMC10653930; doi:10.1128/mbio.01758-23)

Supp. Fig. 1. Soil metabolomic and lipidomic Log2 fold changes with depth and treatment. Volcano plots showing significance (y axis) and magnitude (x axis) of Log2 fold changes in individual compounds faceted by compound class comparing: (i) sampling depth (comparing 0-5 cm and 48-100 cm horizons averaged across all treatments), (ii) irrigation (comparing unirrigated and irrigated bare soils averaged across all depths), and cultivar comparing (iii) irrigated bare soils to irrigated Jose (averaged across all depths) and (iv) irrigated bare soils to irrigated Alkar (averaged across all depths).

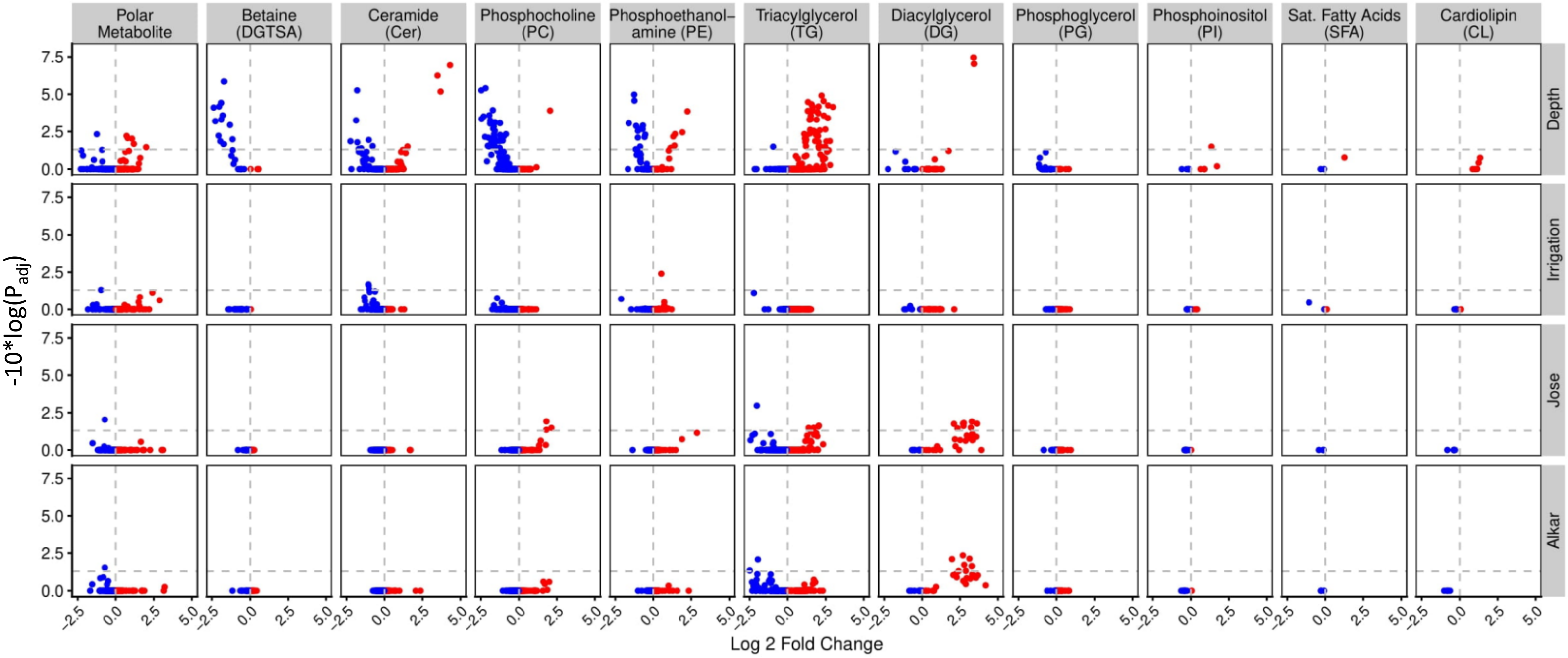

Supplement: Figure S1 — Changes in metabolites. [file mbio.01758-23-s0001.pdf]
