# Supplementary material for: Influence of soil depth, irrigation, and plant genotype on the soil microbiome, metaphenome, and carbon chemistry
Source: mBio. 2023 Sep 20;14(5):e01758-23. doi: 10.1128/mbio.01758-23 (PMC10653930; doi:10.1128/mbio.01758-23)

Supp. Fig. 2. Plots showing magnitude and standard error of class−level significant Log2 fold changes in (a) 16S and (b) ITS ASVs with: sampling depth − comparing 0−5 cm and 48−100 cm horizons averaged across all treatments, irrigation − comparing unirrigated and irrigated bare soils averaged across all depths, and cultivars − comparing irrigated bare soils to irrigated Jose and irrigated Alkar – averaged across all depths.

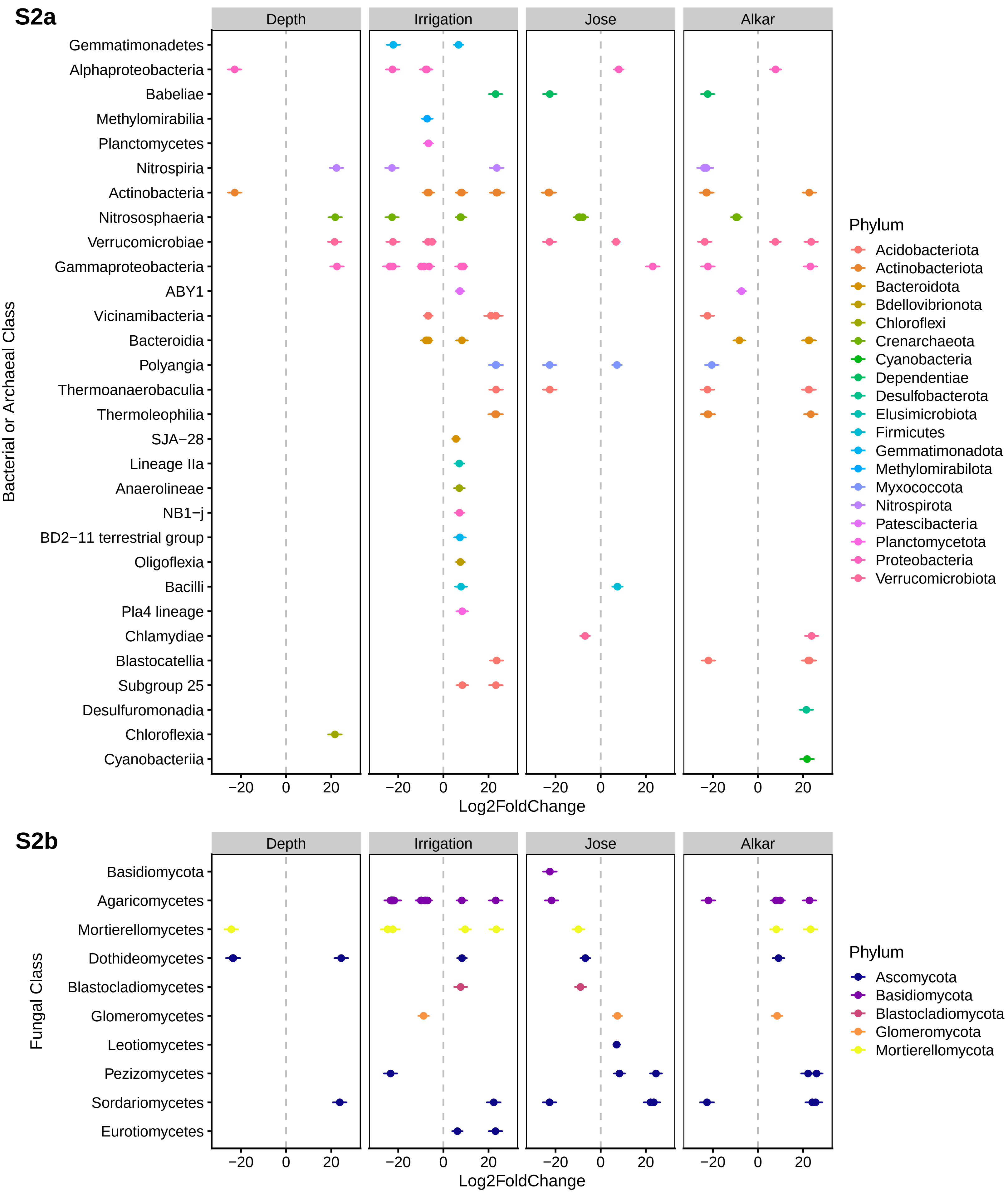

Supplement: Figure S2 — Shifts in taxa. [file mbio.01758-23-s0002.pdf]
