# Supplementary material for: Influence of soil depth, irrigation, and plant genotype on the soil microbiome, metaphenome, and carbon chemistry
Source: mBio. 2023 Sep 20;14(5):e01758-23. doi: 10.1128/mbio.01758-23 (PMC10653930; doi:10.1128/mbio.01758-23)

Supp. Fig. 3. Class-level relative abundance of 16S and ITS soil microbial community composition by (a,c) treatment averaged across all depths and by (b,d) depths averaged across all treatments.

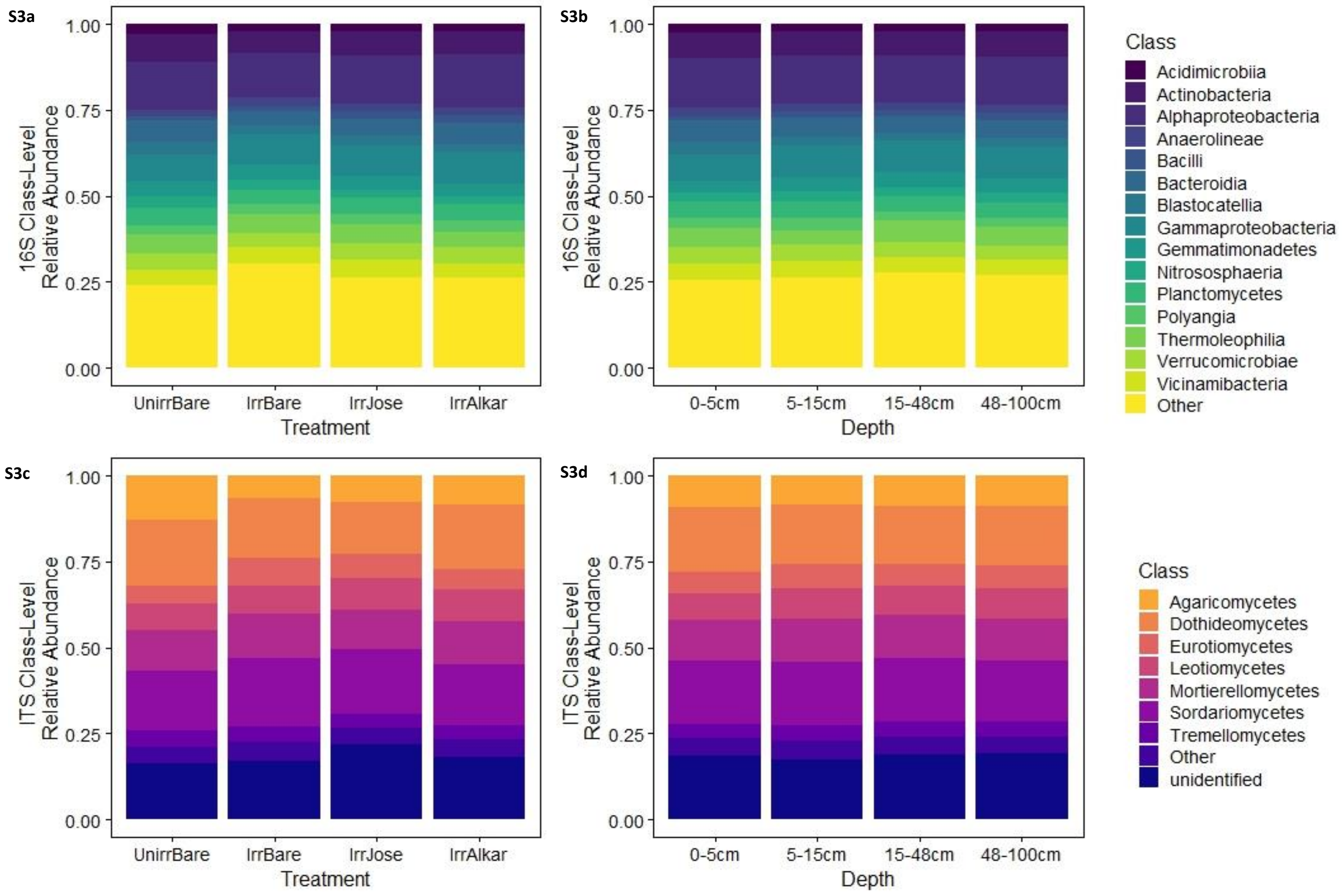

Supplement: Figure S3 — Relative taxon abundance. [file mbio.01758-23-s0003.pdf]
