# Supplementary material for: Influence of soil depth, irrigation, and plant genotype on the soil microbiome, metaphenome, and carbon chemistry
Source: mBio. 2023 Sep 20;14(5):e01758-23. doi: 10.1128/mbio.01758-23 (PMC10653930; doi:10.1128/mbio.01758-23)
Supplement: Table S1 — Abiotic soil properties. [file mbio.01758-23-s0004.pdf]

Supp. Table 1. Mean and standard error (SE) of values of abiotic soil properties in each soil depth increment (averaged across all treatments) and irrigation and plant cultivar treatments (averaged across all soil depth increments in respective treatments)

		**SOM** %		**TOC** %		**POXC** %		**SIC** %		**TN** %		**Moisture** %	
**Treatment**	**Soil depth (cm)**	**Mean**	**SE**	**Mean**	**SE**	**Mean**	**SE**	**Mean**	**SE**	**Mean**	**SE**	**Mean**	**SE**
Mean values for each soil depth increment averaged across all treatments													
	0-5	1.90a	0.07	1.11a	0.04	0.03a	0.00	0.36	0.03	0.14a	0.00	10.0	0.4
	5-15	1.53ab	0.05	1.01a	0.05	0.03b	0.00	0.24	0.02	0.11a	0.00	9.6	0.3
	15-48	1.10bc	0.04	0.41b	0.02	0.02c	0.00	0.44	0.02	0.06b	0.00	8.9	0.3
	48-100	0.77c	0.03	0.26b	0.02	0.02d	0.00	0.43	0.01	0.04b	0.00	9.6	0.3
Mean values for unirrigated bare soils (UB) and irrigated bare soils (IB) averaged across all soil depth increments													
UB		1.37	0.04	1.03a	0.05	0.03	0.00	0.38	0.02	0.12a	0.01	6.1b	0.3
IB		1.20	0.06	0.53b	0.03	0.03	0.00	0.32	0.02	0.07b	0.00	9.9a	0.2
Mean values for irrigated bare soils (IB) and irrigated soils planted with Jose (IJ) averaged across all soil depth increments													
IB		1.20	0.06	0.53	0.03	0.03	0.00	0.32	0.02	0.07	0.00	9.9	0.2
IJ		1.11	0.04	0.55	0.03	0.03	0.00	0.28	0.02	0.07	0.00	11.2	0.3
Mean values for irrigated bare soils (IB) and irrigated soils planted with Alkar (IA) averaged across all soil depth increments													
IB		1.20	0.06	0.53	0.03	0.03	0.00	0.32	0.02	0.07	0.00	9.9	0.2
IA		1.61	0.09	0.69	0.06	0.03	0.00	0.48	0.02	0.09	0.00	11.0	0.2

(Supp. Table 1 continued)

		Soil pH		Ca^{+2} mg kg^{-1}		Mg^{+2} mg kg^{-1}		Na^{+} mg kg^{-1}		K^{+} mg kg^{-1}	
**Treatment**	**Soil depth (cm)**	**Mean**	**SE**	**Mean**	**SE**	**Mean**	**SE**	**Mean**	**SE**	**Mean**	**SE**
Mean values for each soil depth increment averaged across all treatments											
	0-5	7.88c	0.02	2719b	26	230	3	34	2	535a	13
	5-15	7.93bc	0.02	2825b	30	227	3	36	2	435ab	15
	15-48	8.17ab	0.02	3081ab	34	235	4	34	2	363bc	12
	48-100	8.34a	0.02	3269a	23	243	4	43	2	225c	10
Mean values for unirrigated bare soils (UB) and irrigated bare soils (IB) averaged across all soil depth increments											
UB		8.42a	0.02	2988	32	232	3	61a	2	442	17
IB		8.03b	0.02	3026	36	246	5	25b	0	332	13
Mean values for irrigated bare soils (IB) and irrigated Jose (IJ) averaged across all soil depth increments											
IB		8.03	0.02	3026	36	246	5	25	0	332	13
IJ		7.94	0.02	2977	38	237	3	24	1	351	12
Mean values for irrigated bare soils (IB) and irrigated Alkar (IA) averaged across all soil depth increments											
IB		8.03	0.02	3026	36	246	5	25	0	332	13
IA		7.95	0.02	2903	28	220	3	37	2	433	19

(Supp. Table 1 continued)

Treatment	Soil depth (cm)	Olsen P mg kg^{-1} Mean	SE	Sulfur mg kg^{-1} Mean	SE	Zn^{+2} mg kg^{-1} Mean	SE	Mn^{+2} mg kg^{-1} Mean	SE	Fe^{+2} mg kg^{-1} Mean	SE
Mean values for each soil depth increment averaged across all treatments											
	0-5	30a	1	18a	1	1.6a	0.1	14a	1	32a	1
	5-15	27ab	1	14ab	1	1.5ab	0.1	11ab	1	29a	1
	15-48	19bc	1	12ab	0	1.0bc	0.0	6bc	0	22ab	1.4
	48-100	12c	1	10b	0	0.8c	0.0	4c	0	15b	0.5
Mean values for unirrigated bare soils (UB) and irrigated bare soils (IB) averaged across all soil depth increments											
UB		23	1	11	0	0.9	0.0	6	0	23	0.8
IB		22	1	14	1	1.2	0.1	9	1	25	1.1
Mean values for irrigated bare soils (IB) and irrigated Jose (IJ) averaged across all soil depth increments											
IB		22	1	14	1	1.2	0.1	9	1	25	1.1
IJ		21	1	14	1	1.2	0.0	9	1	20	0.9
Mean values for irrigated bare soils (IB) and irrigated Alkar (IA) averaged across all soil depth increments											
IB		22	1	14	1	1.2	0.1	9	1	25	1.1
IA		24	1	16	1	1.6	0.1	12	1	30	1.6
