# Supplementary material for: Influence of soil depth, irrigation, and plant genotype on the soil microbiome, metaphenome, and carbon chemistry
Source: mBio. 2023 Sep 20;14(5):e01758-23. doi: 10.1128/mbio.01758-23 (PMC10653930; doi:10.1128/mbio.01758-23)
Supplement: Table S2 — Polar metabolites. [file mbio.01758-23-s0005.pdf]

Supp. Table 2. Mean and standard error (SE) of log2 normalized relative abundances of polar metabolites in each soil depth increment (averaged across all treatments) and irrigation and plant cultivar treatments (averaged across all soil depth increments in respective treatments)

		**bicarbonate**		**benzoic acid**		**lactic acid**		**myristic acid**		**stearic acid**	
**Treatment**	**Soil depth (cm)**	**Mean**	**SE**	**Mean**	**SE**	**Mean**	**SE**	**Mean**	**SE**	**Mean**	**SE**
Mean Log2 normalized abundance for each soil depth increment averaged across all treatments											
	0-5	1.45b	0.10	2.19b	0.06	1.09c	0.10	0.25b	0.04	1.86b	0.02
	5-15	1.98ab	0.08	2.13b	0.06	1.66bc	0.05	0.28ab	0.03	2.24ab	0.04
	15-48	2.65ab	0.10	2.54ab	0.06	2.24ab	0.09	0.44ab	0.05	2.12ab	0.03
	48-100	2.93a	0.10	2.95a	0.06	3.09a	0.09	0.78a	0.04	2.37a	0.03
Mean Log2 normalized abundance under unirrigated (UB) and irrigated (IB) bare soils averaged across all soil depth increments											
UB		2.53a	0.13	2.99	0.04	2.00	0.05	0.23	0.04	2.25	0.05
IB		1.22b	0.12	2.79	0.04	1.66	0.15	0.77	0.03	2.15	0.03
Mean Log2 normalized abundance under irrigated bare soils (IB) and irrigated Jose (IJ) averaged across all soil depth increments											
IB		1.22b	0.12	2.79a	0.04	1.66	0.15	0.77	0.03	2.15	0.03
IJ		2.87a	0.06	2.03b	0.06	2.21	0.07	0.54	0.04	1.99	0.02
Mean Log2 normalized abundance under irrigated bare soils (IB) and irrigated Alkar (IA) averaged across all soil depth increments											
IB		1.22b	0.12	2.79a	0.04	1.66	0.15	0.77	0.03	2.15	0.03
IA		2.39a	0.03	2.00b	0.07	2.21	0.12	0.20	0.04	2.20	0.04

(Supp. Table 2 continued)

Treatment	Soil depth (cm)	trehalose Mean	trehalose SE	D-mannose Mean	D-mannose SE	L-valine Mean	L-valine SE	urea Mean	urea SE	glucosamine-1-phosphate Mean	glucosamine-1-phosphate SE
Mean Log2 normalized abundance for each soil depth increment averaged across all treatments											
	0-5	2.42a	0.05	1.35	0.13	2.06ab	0.23	2.39	0.16	3.36a	0.06
	5-15	1.97ab	0.06	1.34	0.13	2.77a	0.11	2.82	0.10	3.04a	0.06
	15-48	1.54b	0.05	2.15	0.14	2.14a	0.10	3.09	0.07	2.80a	0.06
	48-100	0.68c	0.08	2.15	0.22	-0.07b	0.19	3.46	0.10	1.20b	0.15
Mean Log2 normalized abundance under unirrigated (UB) and irrigated bare soils (IB) averaged across all soil depth increments											
UB		2.50a	0.07	-0.01b	0.23	0.49	0.19	3.66a	0.08	3.46	0.08
IB		1.58b	0.06	2.88a	0.07	1.68	0.21	2.30b	0.15	2.36	0.15
Mean Log2 normalized abundance under irrigated bare soils (IB) and irrigated Jose (IJ) averaged across all soil depth increments											
IB		1.58	0.06	2.88	0.07	1.68	0.21	2.30	0.15	2.36	0.15
IJ		1.48	0.05	1.92	0.09	2.60	0.11	3.35	0.06	2.68	0.05
Mean Log2 normalized abundance under irrigated bare soils (IB) and irrigated Alkar (IA) averaged across all soil depth increments											
IB		1.58	0.06	2.88	0.07	1.68	0.21	2.30	0.15	2.36	0.15
IA		1.09	0.09	2.21	0.08	2.12	0.19	2.45	0.10	1.90	0.12

(Supp. Table 2 continued)

		D-arabitol		D-mannitol		alloinositol		myoinositol		campesterol	
**Treatment**	**Soil depth (cm)**	**Mean**	**SE**	**Mean**	**SE**	**Mean**	**SE**	**Mean**	**SE**	**Mean**	**SE**
Mean Log2 normalized abundance for each soil depth increment averaged across all treatments											
	0-5	0.52a	0.18	4.67a	0.08	1.12a	0.04	3.05a	0.05	0.82a	0.04
	5-15	-0.39ab	0.15	3.9ab	0.06	1.28a	0.07	2.82a	0.06	0.59a	0.07
	15-48	-0.99ab	0.17	3.43bc	0.06	0.91ab	0.07	2.55a	0.06	-0.19b	0.04
	48-100	-1.78b	0.16	2.41c	0.14	0.22b	0.05	1.80b	0.05	-1.66c	0.08
Mean Log2 normalized abundance under unirrigated (UB) and irrigated bare soils (IB) averaged across all soil depth increments											
UB		0.30	0.17	4.02	0.10	0.59	0.07	2.75	0.07	-0.03	0.08
IB		-1.53	0.21	3.39	0.12	1.27	0.07	2.35	0.09	-0.29	0.08
Mean Log2 normalized abundance under irrigated bare soils (IB) and irrigated Jose (IJ) averaged across all soil depth increments											
IB		-1.53	0.21	3.39	0.12	1.27	0.07	2.35	0.09	-0.29	0.08
IJ		-0.57	0.15	3.84	0.05	0.93	0.06	2.66	0.06	-0.04	0.12
Mean Log2 normalized abundance under irrigated bare soils (IB) and irrigated Alkar (IA) averaged across all soil depth increments											
IB		-1.53	0.21	3.39	0.12	1.27	0.07	2.35	0.09	-0.29	0.08
IA		-0.85	0.15	3.15	0.15	0.74	0.06	2.46	0.06	0.18	0.11

(Supp. Table 2 continued)

Treatment	Soil depth (cm)	capric acid		lauric acid		nonanoic acid		tagatose		D-fructose	
		**Mean**	**SE**	**Mean**	**SE**	**Mean**	**SE**	**Mean**	**SE**	**Mean**	**SE**
Mean Log2 normalized abundance for each soil depth increment averaged across all treatments											
	0-5	1.71	0.05	2.47	0.05	2.47	0.05	4.24	0.19	3.44	0.21
	5-15	1.54	0.05	2.79	0.05	2.35	0.05	3.96	0.20	3.31	0.20
	15-48	1.30	0.05	2.72	0.04	2.41	0.04	4.28	0.21	3.65	0.21
	48-100	1.64	0.08	2.83	0.05	2.84	0.04	3.09	0.26	2.53	0.24
Mean Log2 normalized abundance under unirrigated (UB) and irrigated bare soils (IB) averaged across all soil depth increments											
UB		1.80	0.04	2.96	0.04	2.63	0.04	3.46	0.14	2.49	0.16
IB		1.95	0.05	3.10	0.03	2.94	0.04	1.94	0.31	1.39	0.28
Mean Log2 normalized abundance under irrigated bare soils (IB) and irrigated Jose (IJ) averaged across all soil depth increments											
IB		1.95	0.05	3.10a	0.03	2.94a	0.04	1.94b	0.31	1.39b	0.28
IJ		1.52	0.05	2.38b	0.03	2.37b	0.05	5.06a	0.15	4.43a	0.15
Mean Log2 normalized abundance under irrigated bare soils (IB) and irrigated Alkar (IA) averaged across all soil depth increments											
IB		1.95a	0.05	3.10a	0.03	2.94a	0.04	1.94b	0.31	1.39b	0.28
IA		0.93b	0.06	2.38b	0.04	2.12b	0.05	5.11a	0.08	4.62a	0.08
