# Supplementary material for: Influence of soil depth, irrigation, and plant genotype on the soil microbiome, metaphenome, and carbon chemistry
Source: mBio. 2023 Sep 20;14(5):e01758-23. doi: 10.1128/mbio.01758-23 (PMC10653930; doi:10.1128/mbio.01758-23)
Supplement: Table S3 — Protein abundances. [file mbio.01758-23-s0006.pdf]

Supp. Table 3. Mean and standard error (SE) of log2 normalized relative abundances of proteins in each soil depth increment (averaged across all treatments) and irrigation and plant cultivar treatments (averaged across all soil depth increments in respective treatments)

		**ribose ABC transport**		**multiple-sugar ABC transport**		**Glucosyl-ceramidase**		**Polar amino acid ABC transport**		**L-amino acid ABC transport**		**alpha-glucoside ABC transport**	
**Treatment**	**Soil depth (cm)**	**Mean**	**SE**	**Mean**	**SE**	**Mean**	**SE**	**Mean**	**SE**	**Mean**	**SE**	**Mean**	**SE**
Mean Log2 normalized abundance for each soil depth increment averaged across all treatments													
	0-5	299a	23	172a	7	168ab	7	54bc	3	137b	8	166b	8
	5-15	672a	19	110ab	6	217a	7	35c	2	183b	8	144b	8
	15-48	582a	29	77b	6	112bc	7	189a	16	239a	16	327a	21
	48-100	210b	12	28b	3	35c	2	153ab	12	231a	17	273ab	17
Mean Log2 normalized abundance under unirrigated (UB) and irrigated bare soils (IB) averaged across all soil depth increments													
UB		428	21	131	7	100	7	90	10	212	12	275	13
IB		550	26	82	8	196	10	100	9	101	7	149	16
Mean Log2 normalized abundance under irrigated bare soils (IB) and irrigated Jose (IJ) averaged across all soil depth increments													
IB		550	26	82	8	196	10	100	9	101	7	149	16
IJ		436	29	83	8	129	7	148	12	221	16	264	17
Mean Log2 normalized abundance under irrigated bare soils (IB) and irrigated Alkar (IA) averaged across all soil depth increments													
IB		550	26	82	8	196	10	100	9	101	7	149	16
IA		429	33	92	6	154	7	94	14	232	12	192	14

(Supp. Table 3 continued)

Treatment	Soil depth (cm)	Superoxide dismutase		Plant pathogen interaction		Heat shock		Methane hydrolase		F-type ATPase (alpha)		F-type ATPase (beta)		Histone H4	
		Mean	SE	Mean	SE	Mean	SE	Mean	SE	Mean	SE	Mean	SE	Mean	SE
Mean Log2 normalized abundance for each soil depth increment averaged across all treatments															
	0-5	217ab	10	1103a	95	109a	4	214a	13	117ab	10	308a	21	32ab	2
	5-15	230a	10	1103ab	72	86ab	4	292a	15	152a	6	221ab	9	35a	2
	15-48	104bc	8	812bc	48	68b	4	301a	12	95ab	6	132bc	9	35ab	2
	48-100	77c	7	165c	9	49b	2	108b	6	55b	5	83c	5	32b	1
Mean Log2 normalized abundance under unirrigated (UB) and irrigated bare soils (IB) averaged across all soil depth increments															
UB		150	11	1232	85	97	4	276	14	126	8	170	10	25	1
IB		235	8	834	52	65	4	193	13	74	4	228	19	25	2
Mean Log2 normalized abundance under irrigated bare soils (IB) and irrigated Jose (IJ) averaged across all soil depth increments															
IB		235	8	834	52	65	4	193	13	74	4	228	19	25b	2
IJ		149	10	661	68	78	4	247	13	124	7	251	14	39a	2
Mean Log2 normalized abundance under irrigated bare soils (IB) and irrigated Alkar (IA) averaged across all soil depth increments															
IB		35	8	834	52	65	4	193	13	74	4	228	19	25b	2
IA		162	12	682	60	89	5	246	14	115	10	149	14	45a	2
