# Supplementary material for: Influence of soil depth, irrigation, and plant genotype on the soil microbiome, metaphenome, and carbon chemistry
Source: mBio. 2023 Sep 20;14(5):e01758-23. doi: 10.1128/mbio.01758-23 (PMC10653930; doi:10.1128/mbio.01758-23)
Supplement: Table S4 — Significant differences in metabolites, proteins and lipids. [file mbio.01758-23-s0007.pdf]

Supp. Table 4. Significant Log2 Fold changes (values for fold change) or significant differences in ANOVA (+ or - for fold change) in normalized relative abundance of polar metabolites, proteins, and lipids identified in all 48 soil samples. Abundance was tested for differences between depth (comparing top (0-5 cm) and bottom (48-100 cm) horizons), irrigation (comparing unirrigated bare to irrigated bare soils), and cultivar (comparing irrigated bare soils to irrigated Alkar and Jose individually).

Class	Factor	Compound	CO, KO, Lipid abbrev.	fold change	P_{adj}
Polar metabolite	Depth	Unknown 026		0.71	0.01
Polar metabolite	Depth	Unknown 022		0.79	0.01
Polar metabolite	Depth	Unknown 044		1.07	0.01
Polar metabolite	Depth	Unknown 042		1.19	0.02
Polar metabolite	Depth	lactic acid	C00249	2.00	0.04
Polar metabolite	Depth	bicarbonate	C02522	+	0.01
Polar metabolite	Depth	benzoic acid	C01571	+	0.01
Polar metabolite	Depth	myristic acid	C01595	+	0.05
Polar metabolite	Depth	palmitic acid	C00249	+	0.05
Polar metabolite	Depth	stearic acid	C01530	+	0.05
Polar metabolite	Depth	glucosamine-1-phosphate	C00645	-	0.01
Polar metabolite	Depth	L-valine	C00183	-	0.01
Polar metabolite	Depth	trehalose	C00392	-	0.01
Polar metabolite	Depth	D-arabitol	C01235	-	0.05
Polar metabolite	Depth	D-mannitol	C00116	-	0.01
Polar metabolite	Depth	campesterol	C00156	-	0.05
Polar metabolite	Depth	alloinositol	C00160	-	0.01
Polar metabolite	Depth	myoinositol	C01432	-1.25	0.01
Polar metabolite	Irrigation	bicarbonate	C02522	-	0.04
Polar metabolite	Irrigation	urea	C00086	-	0.03
Polar metabolite	Irrigation	trehalose	C00392	-	0.01
Polar metabolite	Irrigation	D-mannose	C00181	+	0.01
Polar metabolite	Irrigation	Unknown 078		-0.97	0.05
Polar metabolite	Alkar	bicarbonate	C02522	+	0.01
Polar metabolite	Alkar	benzoic acid	C01571	-	0.01

(Supp. Table 4 continued)

Class	Factor	Compound	CO, KO, Lipid abbrev.	fold change	P_{adj}
Polar metabolite	Alkar	lauric acid	C02679	-0.11	0.03
Polar metabolite	Alkar	nonanoic acid	C01601	-	0.01
Polar metabolite	Alkar	D-fructose	C00031	+	0.01
Polar metabolite	Alkar	tagatose	C01971	+	0.01
Polar metabolite	Alkar	capric acid	C00137	-	0.01
Polar metabolite	Alkar	Unknown 029		-0.49	0.01
Polar metabolite	Jose	bicarbonate	C02522	+	0.01
Polar metabolite	Jose	benzoic acid	C01571	-	0.01
Polar metabolite	Jose	lauric acid	C02679	-0.11	0.01
Polar metabolite	Jose	nonanoic acid	C01601	-	0.01
Polar metabolite	Jose	D-fructose	C00031	+	0.01
Polar metabolite	Jose	tagatose	C01971	+	0.02
Protein	Depth	Polar amino acid ABC transport	K02030	+	0.05
Protein	Depth	L-amino acid ABC transport	K09969	+	0.05
Protein	Depth	Alpha-glucoside ABC transport	K10232	+	0.05
Protein	Depth	Multiple sugar ATP-binding ABC transport	K10112	-	0.05
Protein	Depth	Glucosylceramidase GBA	K01201	-	0.05
Protein	Depth	Superoxide dismutase, Fe-Mn	K04564	-	0.05
Protein	Depth	F-type ATPase	K02112	-	0.05
Protein	Depth	Ribose ABC transport	K10439	-	0.05
Protein	Depth	Histone H4	K11254	-	0.05
Protein	Depth	Plant pathogen elongation/interaction	K02358	-	0.05
Protein	Depth	Heat shock	K03283	-	0.05
Protein	Depth	Methane metabolism hydrolase Fae	K10713	-	0.05
Protein	Alkar	Histone H4	K11254	+	0.05
Protein	Alkar	F-type ATPase	K02111	+	0.05

(Supp. Table 4 continued)

Class	Factor	Compound	CO, KO, Lipid abbrev.	fold change	P_{adj}
Cardiolipin	Depth	CL(17:1/17:1/17:1/19:1)	CL(72:4)	+	0.00
Phosphoglycerol	Depth	PG(16:0/18:1)	PG(34:1)	-	0.03
Phosphoinositol	Depth	PI(22:0/22:0)	PI(44:0)	1.36	0.03
Saturated	Depth	Hydroxyphthioceranic Acid (33:0)	Hyd(33:0)	+	0.00
Betaine	Depth	DGTSA(15:0/16:1)	DGTSA(31:1)	-2.38	0.00
Betaine	Depth	DGTSA(16:1/16:1)	DGTSA(32:2)	-2.27	0.00
Betaine	Depth	DGTSA(16:0/18:2)	DGTSA(34:2)	-2.06	0.01
Betaine	Depth	DGTSA(15:0/18:0)	DGTSA(33:0)	-2.02	0.00
Betaine	Depth	DGTSA(15:0/18:1)_A	DGTSA(33:1)_A	-1.94	0.01
Betaine	Depth	DGTSA(16:0/16:1); DGTSA(15:0/17:1)_A	DGTSA(32:1)_A	-1.89	0.00
Betaine	Depth	DGTSA(16:0/16:1); DGTSA(15:0/17:1)_B	DGTSA(32:1)_B	-1.89	0.00
Betaine	Depth	DGTSA(16:0/16:1); DGTSA(15:0/17:1)_C	DGTSA(32:1)_C	-1.88	0.00
Betaine	Depth	DGTSA(15:0/18:1)_B	DGTSA(33:1)_B	-1.78	0.00
Betaine	Depth	DGTSA(16:0/18:0)_A; DGTSA(17:0/17:0)	DGTSA(34:0)	-1.73	0.02
Betaine	Depth	DGTSA(16:0/18:0)_B	DGTSA(34:0)_B	-1.71	0.00
Betaine	Depth	DGTSA(16:0/16:0)	DGTSA(32:0)	-1.34	0.00
Betaine	Depth	DGTSA(16:0/19:1)	DGTSA(35:1)	-1.17	0.01
Ceramides	Depth	Cer(d16:1/18:1(2OH))_C	Cer(d34:2)-2OH_C	-2.77	0.01
Ceramides	Depth	Cer(d19:1/18:2)_B	Cer(d37:3)_B	-2.24	0.01
Ceramides	Depth	Cer(d18:2/18:1)_B	Cer(d36:3)_B	-1.87	0.00
Ceramides	Depth	Cer(d18:1/18:1)	Cer(d36:2)	-1.80	0.00
Ceramides	Depth	Cer(d18:1/16:0(2OH))_A	Cer(d34:1)-2OH_A	-1.79	0.02

(Supp. Table 4 continued)

Class	Factor	Compound	CO, KO, Lipid abbrev.	fold change	P_{adj}
Ceramides	Depth	Cer(d18:2/20:1)_B	Cer(d38:3)_B	-1.70	0.05
Ceramides	Depth	Cer(d18:0/20:0(2OH))_A	Cer(d38:0)-2OH_A	-1.53	0.04
Ceramides	Depth	Cer(d18:1/16:0)_B	Cer(d34:1)_B	-1.04	0.01
Ceramides	Depth	Cer(d19:1/18:2)_A	Cer(d37:3)_A	-0.78	0.03
Ceramides	Depth	Cer(d18:0/17:0)_B	Cer(d35:0)_B	1.51	0.03
Ceramides	Depth	Cer(d20:0/16:0)_A; Cer(d18:0/18:0)_A	Cer(d36:0)	3.49	0.00
Ceramides	Depth	Cer(d18:0/18:0)_A	Cer(d36:0)_A	3.69	0.00
Ceramides	Depth	GalCer(d18:1/16:1)	GalCer(d34:2)	3.98	0.00
Ceramides	Depth	Cer(d18:0/17:0)_A	Cer(d35:0)_A	4.31	0.00
Ceramides	Depth	Cer(d18:0/16:0)_A	Cer(d34:0)_A	5.33	0.00
Ceramides	Depth	Cer(t18:0/16:0(2OH))_A	Cer(t34:0)-2OH_A	-	0.01
Ceramides	Irrigation	Cer(d18:0/17:0)_C	Cer(d35:0)_C	-1.06	0.02
Ceramides	Irrigation	Cer(t18:0/18:0(2OH))_B	Cer(t36:0)-2OH_B	-1.04	0.03
Ceramides	Irrigation	Cer(d18:0/16:0(2OH))_A	Cer(d34:0)-2OH_A	-1.01	0.04
Phosphoethanolamine	Depth	PE(O-15:0/15:1)	PE(O-30:1)	-1.61	0.00
Phosphoethanolamine	Depth	PE(P-16:0/16:1)_B	PE(P-32:1)_B	-1.26	0.00
Phosphoethanolamine	Depth	PE(P-16:0/15:0)	PE(P-31:0)	-1.24	0.00
Phosphoethanolamine	Depth	PE(18:1/20:1)_A	PE(38:2)_A	-1.15	0.05
Phosphoethanolamine	Depth	PE(17:1/18:1)_B	PE(35:2)_B	-1.13	0.00
Phosphoethanolamine	Depth	PE(16:1/17:1)_A	PE(33:2)_A	-1.01	0.01

(Supp. Table 4 continued)

Class	Factor	Compound	CO, KO, Lipid abbrev.	fold change	P_{adj}
Phosphoethanolamine	Depth	PE(16:1/17:1)_B	PE(33:2)_B	-1.01	0.00
Phosphoethanolamine	Depth	PE(16:1/16:1)_A	PE(32:2)_A	-0.87	0.03
Phosphoethanolamine	Depth	PE(O-15:0/15:0)	PE(O-30:0)	-0.86	0.00
Phosphoethanolamine	Depth	PE(16:1/17:1)_C	PE(33:2)_C	-0.70	0.00
Phosphoethanolamine	Depth	PE(15:0/16:1)_A	PE(31:1)_A	-0.68	0.00
Phosphoethanolamine	Depth	PE(15:0/15:0)	PE(30:0)	-0.57	0.01
Phosphoethanolamine	Depth	PE(17:1/18:1)_C	PE(35:2)_C	-0.53	0.01
Phosphoethanolamine	Depth	PE(17:0/18:0)_B	PE(35:0)_B	1.13	0.04
Phosphoethanolamine	Depth	PE(19:1/19:1)_B	PE(38:2)_B	1.31	0.01
Phosphoethanolamine	Depth	PE(17:0/17:0)	PE(34:0)	1.39	0.03
Phosphoethanolamine	Depth	PE(16:0/17:0)_C	PE(33:0)_C	1.43	0.00
Phosphoethanolamine	Depth	PE(P-20:0/17:0)	PE(P-37:0)	1.91	0.00
Phosphoethanolamine	Depth	PE(15:0/18:0)_A; PE(16:0/17:0)_A	PE(33:0)_A	2.25	0.00
Phosphoethanolamine	Depth	PE(17:1/17:1)_A	PE(34:2)_A	-	0.02
Phosphoethanolamine	Depth	PE(15:0/15:1)_B; PE(14:0/16:1)_B	PE(30:1)_B	-	0.03
Phosphoethanolamine	Depth	PE(15:0/16:0)_A	PE(31:0)_A	+	0.01
Phosphoethanolamine	Irrigation	PE(15:0/16:0)_B	PE(31:0)_B	0.53	0.00
Phosphocholine	Depth	PC(O-16:0/14:0)	PC(O-30:0)	-2.47	0.00
Phosphocholine	Depth	PC(16:1/16:1)_A	PC(32:2)	-2.46	0.00
Phosphocholine	Depth	PC(15:0/16:1)_B	PC(31:1)	-2.32	0.00
Phosphocholine	Depth	PC(16:1/20:4)	PC(36:5)	-2.23	0.01
Phosphocholine	Depth	PC(20:4/20:4)	PC(40:8)	-2.20	0.00
Phosphocholine	Depth	PC(15:1/16:1)_A	PC(31:2)_A	-2.04	0.03
Phosphocholine	Depth	PC(14:1/16:0)_A	PC(30:1)_A	-2.01	0.01
Phosphocholine	Depth	PC(16:1/16:1)_C	PC(32:2)_C	-1.97	0.03

(Supp. Table 4 continued)

Class	Factor	Compound	CO, KO, Lipid abbrev.	fold change	P_{adj}
Phosphocholine	Depth	PC(20:5/20:5)	PC(40:10)	-1.95	0.00
Phosphocholine	Depth	PC(16:1/17:1)_A	PC(33:2)	-1.89	0.00
Phosphocholine	Depth	PC(15:0/16:1)_A	PC(31:1)_A	-1.88	0.00
Phosphocholine	Depth	PC(O-16:0/15:0)	PC(O-31:0)	-1.87	0.01
Phosphocholine	Depth	PC(16:1/17:1)_B	PC(33:2)_B	-1.84	0.00
Phosphocholine	Depth	PC(16:1/18:2)_A	PC(34:3)	-1.81	0.02
Phosphocholine	Depth	PC(18:1/19:1)_A	PC(37:2)	-1.71	0.00
Phosphocholine	Depth	PC(15:0/15:0)_A	PC(30:0)_A	-1.69	0.00
Phosphocholine	Depth	PC(O-17:0/17:0)	PC(O-34:0)	-1.66	0.04
Phosphocholine	Depth	PC(18:1/18:1)_B	PC(36:2)_B	-1.65	0.00
Phosphocholine	Depth	PC(O-14:0/16:1)_A	PC(O-30:1)_A	-1.64	0.00
Phosphocholine	Depth	PC(16:1/18:1)_A	PC(34:2)_A	-1.63	0.01
Phosphocholine	Depth	PC(16:0/20:4)	PC(36:4)	-1.60	0.03
Phosphocholine	Depth	PC(20:4/20:5)	PC(40:9)	-1.58	0.00
Phosphocholine	Depth	PC(16:0/16:1)_B	PC(32:1)_B	-1.58	0.00
Phosphocholine	Depth	PC(18:1/18:1)_A	PC(36:2)_A	-1.54	0.01
Phosphocholine	Depth	PC(18:1/19:1)_B	PC(37:2)_B	-1.33	0.01
Phosphocholine	Depth	PC(16:0/16:1)_A; PC(15:0/17:1)_B	PC(32:1)	-1.28	0.04
Phosphocholine	Depth	PC(20:1/20:1)	PC(40:2)	-1.26	0.04
Phosphocholine	Depth	PC(15:0/16:0)_B	PC(31:0)_B	-1.24	0.00
Phosphocholine	Depth	PC(16:1/16:1)_E	PC(32:2)_E	-1.23	0.01
Phosphocholine	Depth	PC(14:1/15:1)	PC(29:2)	-1.23	0.01
Phosphocholine	Depth	PC(15:0/20:1)	PC(35:1)	-1.21	0.01
Phosphocholine	Depth	PC(17:1/18:1); PC(16:1/19:1)	PC(35:2)	-1.13	0.01
Phosphocholine	Depth	PC(16:0/17:1)_B	PC(33:1)	-1.07	0.01

(Supp. Table 4 continued)

Class	Factor	Compound	CO, KO, Lipid abbrev.	fold change	P_{adj}
Phosphocholine	Depth	PC(16:0/18:1)_B	PC(34:1)	-1.00	0.01
Phosphocholine	Depth	PC(18:0/18:1)	PC(36:1)	-0.85	0.00
Phosphocholine	Depth	PC(15:0/15:0)_B	PC(30:0)_B	2.05	0.00
Phosphocholine	Depth	PC(13:0/18:0)	PC(31:0)	3.77	0.00
Phosphocholine	Depth	PC(16:1/17:0)_A	PC(33:1)_A	-	0.02
Phosphocholine	Depth	PC(14:1/16:1)	PC(30:2)	-	0.04
Phosphocholine	Irrigation	PC(18:1/20:4)_A	PC(38:5)	1.81	0.01
Phosphocholine	Irrigation	PC(18:1/20:5)	PC(38:6)	1.80	0.04
Phosphocholine	Irrigation	PC(18:3/18:3)_B; PC(16:1/20:5)	PC(36:6)	2.14	0.03
Diacylglycerol	Depth	DG(16:0/18:0/0:0)	DG(34:0)	3.38	0.00
Diacylglycerol	Depth	DG(18:0/0:0/18:0)	DG(36:0)	3.42	0.00
Diacylglycerol	Depth	DG(19:1/21:0/0:0)	DG(40:1)	+	0.02
Diacylglycerol	Alkar	DG(16:0/18:3/0:0)	DG(34:3)	3.13	0.01
Diacylglycerol	Alkar	DG(16:1/18:3/0:0)	DG(34:4)	3.28	0.02
Diacylglycerol	Alkar	DG(18:1/18:2/0:0)_B	DG(36:3)	2.78	0.02
Diacylglycerol	Alkar	DG(18:1/18:3/0:0)	DG(36:4)	2.69	0.00
Diacylglycerol	Alkar	DG(18:1/20:3/0:0)	DG(38:4)	2.41	0.05
Diacylglycerol	Alkar	DG(18:2/18:3/0:0)	DG(36:5)	1.97	0.01
Diacylglycerol	Alkar	DG(18:1/20:4/0:0)	DG(38:5)	2.88	0.05
Diacylglycerol	Jose	DG(16:0/18:3/0:0)	DG(34:3)	3.29	0.01
Diacylglycerol	Jose	DG(16:1/18:3/0:0)	DG(34:4)	3.60	0.02
Diacylglycerol	Jose	DG(18:1/18:2/0:0)_B	DG(36:3)	2.73	0.02
Diacylglycerol	Jose	DG(18:1/18:3/0:0)	DG(36:4)	2.69	0.02
Diacylglycerol	Jose	DG(18:1/20:3/0:0)	DG(38:4)	2.75	0.02
Diacylglycerol	Jose	DG(18:2/18:3/0:0)	DG(36:5)	2.10	0.02
Diacylglycerol	Jose	DG(16:0/18:1/0:0)	DG(34:1)	2.33	0.04

(Supp. Table 4 continued)

Class	Factor	Compound	CO, KO, Lipid abbrev.	fold change	P_{adj}
Diacylglycerol	Jose	DG(16:1/24:1)	DG(40:2)	3.22	0.03
Triacylglycerol	Depth	TG(18:2/18:2/20:4)_B; TG(16:0/20:4/20:4)_B	TG(56:8)_B	-0.96	0.03
Triacylglycerol	Depth	TG(18:1/18:1/18:3); TG(18:1/18:2/18:2); TG(16:1/18:1/20:3)	TG(54:5)	1.09	0.00
Triacylglycerol	Depth	TG(16:1/18:1/18:1)_B; TG(16:0/18:1/18:2)_B	TG(52:3)_B	1.13	0.01
Triacylglycerol	Depth	TG(16:0/16:1/17:1); TG(15:0/17:1/17:1)_B	TG(49:2)	1.16	0.01
Triacylglycerol	Depth	TG(16:1/17:0/18:2); TG(15:1/18:1/18:1); TG(16:1/17:1/18:1); TG(17:1/17:1/17:1); TG(16:1/17:0/18:2)	TG(51:3)	1.16	0.02
Triacylglycerol	Depth	TG(17:1/18:1/18:1); TG(17:1/17:1/19:1); TG(16:0/18:2/19:1); TG(16:1/18:1/19:1)	TG(53:3)	1.17	0.02
Triacylglycerol	Depth	TG(16:0/18:1/18:1)	TG(52:2)	1.20	0.03
Triacylglycerol	Depth	TG(18:0/18:1/18:1); TG(17:0/18:1/19:1); TG(16:0/16:1/22:1)	TG(54:2)	1.22	0.01
Triacylglycerol	Depth	TG(18:1/18:1/18:1)	TG(54:3)	1.27	0.01
Triacylglycerol	Depth	TG(16:0/18:0/18:1); TG(17:0/17:0/18:1)	TG(52:1)	1.33	0.00
Triacylglycerol	Depth	TG(17:0/17:1/17:1); TG(15:0/18:1/18:1); TG(16:1/17:0/18:1); TG(16:0/17:1/18:1)	TG(51:2)	1.34	0.00
Triacylglycerol	Depth	TG(18:1/18:1/18:2)	TG(54:4)	1.44	0.00
Triacylglycerol	Depth	TG(16:0/16:0/18:1)_B	TG(50:1)_B	1.48	0.00

(Supp. Table 4 continued)

**Class**	**Factor**	**Compound**	**CO, KO, Lipid abbrev.**	**fold change**	**P_{adj}**
Triacylglycerol	Depth	TG(15:1/15:1/17:0)_A; TG(15:0/16:1/16:1)_A; TG(15:0/15:0/17:2)	TG(47:2)	1.48	0.00
Triacylglycerol	Depth	TG(15:0/16:0/16:1)_B; TG(15:0/15:1/17:0); TG(14:0/16:1/17:0)	TG(47:1)_B	1.52	0.00
Triacylglycerol	Depth	TG(15:0/16:0/18:1)	TG(49:1)	1.53	0.00
Triacylglycerol	Depth	TG(17:0/18:1/18:1); TG(15:0/19:1/19:1); TG(16:0/18:1/19:1)	TG(53:2)	1.57	0.00
Triacylglycerol	Depth	TG(16:0/18:0/18:0)_A	TG(52:0)_A	1.62	0.00
Triacylglycerol	Depth	TG(15:0/17:1/17:1)_A	TG(49:2)_A	1.64	0.00
Triacylglycerol	Depth	TG(16:0/16:0/19:1); TG(15:0/18:0/18:1)	TG(51:1)	1.65	0.00
Triacylglycerol	Depth	TG(15:0/15:0/16:0)	TG(46:0)	1.72	0.00
Triacylglycerol	Depth	TG(14:1/15:0/17:1)	TG(46:2)	1.81	0.01
Triacylglycerol	Depth	TG(17:0/17:0/19:0)	TG(53:0)	1.86	0.01
Triacylglycerol	Depth	TG(16:0/16:0/17:0)_B	TG(49:0)_B	1.90	0.00
Triacylglycerol	Depth	TG(16:0/16:1/18:0)_A	TG(50:1)_A	1.95	0.00
Triacylglycerol	Depth	TG(14:1/16:0/16:0)_A; TG(15:0/15:0/16:1)_B; TG(14:0/15:0/17:1)_B;TG(14:0/16:0/16:1)_B	TG(46:1)_B	1.99	0.01
Triacylglycerol	Depth	TG(15:0/16:0/16:0)_B; TG(15:0/15:0/17:0)_B	TG(47:0)	2.01	0.00
Triacylglycerol	Depth	TG(15:0/17:0/18:0); TG(14:0/18:0/18:0); TG(16:0/17:0/17:0)_A	TG(50:0)	2.02	0.00

(Supp. Table 4 continued)

**Class**	**Factor**	**Compound**	**CO, KO, Lipid abbrev.**	**fold change**	P_{adj}
Triacylglycerol	Depth	TG(15:0/15:0/16:1)_A; TG(14:0/14:0/18:1); TG(14:0/15:0/17:1)_A; TG(14:0/16:0/16:1)_A	TG(46:1)_A	2.03	0.02
Triacylglycerol	Depth	TG(15:0/16:0/16:0)_A; TG(15:0/15:0/17:0)_A	TG(47:0)_A	2.04	0.03
Triacylglycerol	Depth	TG(13:0/14:0/18:1); TG(14:0/15:0/16:1); TG(13:0/16:0/16:1)	TG(45:1)	2.05	0.04
Triacylglycerol	Depth	TG(16:0/16:0/16:1)_B	TG(48:1)_B	2.10	0.00
Triacylglycerol	Depth	TG(14:0/14:0/16:0)_A	TG(44:0)_A	2.18	0.03
Triacylglycerol	Depth	TG(16:0/17:0/20:1); TG(16:1/18:0/19:0)	TG(53:1)	2.22	0.00
Triacylglycerol	Depth	TG(16:0/17:0/17:0)_B; TG(16:0/16:0/18:0)_B	TG(50:0)_B	2.35	0.00
Triacylglycerol	Depth	TG(16:0/16:0/18:0)_A	TG(50:0)_A	2.37	0.00
Triacylglycerol	Depth	TG(14:0/14:0/16:0)_B	TG(44:0)_B	2.46	0.03
Triacylglycerol	Depth	TG(17:0/17:0/17:0)	TG(51:0)	2.47	0.00
Triacylglycerol	Depth	TG(15:0/16:0/16:1)_A; TG(14:0/16:0/17:1)	TG(47:1)	2.54	0.01
Triacylglycerol	Depth	TG(16:0/16:0/17:0)_A; TG(15:0/15:0/19:0); TG(15:0/17:0/17:0); TG(15:0/16:0/18:0)	TG(49:0)	2.63	0.00
Triacylglycerol	Depth	TG(16:0/18:0/18:0)_B	TG(52:0)_B	2.65	0.00
Triacylglycerol	Depth	TG(13:0/15:0/15:0)	TG(43:0)	2.77	0.01
Triacylglycerol	Depth	TG(16:0/16:0/16:0)	TG(48:0)	2.97	0.00
Triacylglycerol	Depth	TG(16:1/18:1/18:2); TG(16:0/18:2/18:2)	TG(52:4)	+	0.01

(Supp. Table 4 continued)

**Class**	**Factor**	**Compound**	**CO, KO, Lipid abbrev.**	**fold change**	**P_{adj}**
Triacylglycerol	Depth	TG(13:0/14:1/14:1)	TG(41:2)	+	0.01
Triacylglycerol	Depth	TG(12:0/14:1/16:1); TG(13:0/14:1/15:1)	TG(42:2)	+	0.03
Triacylglycerol	Depth	TG(12:0/12:0/13:0)	TG(37:0)	+	0.04
Triacylglycerol	Alkar	TG(17:0/20:4/20:4)	TG(57:8)	-1.96	0.01
Triacylglycerol	Alkar	TG(14:0/15:0/16:0)	TG(45:0)	-2.49	0.05
Triacylglycerol	Jose	TG(17:0/20:4/20:4)	TG(57:8)	-2.03	0.00
Triacylglycerol	Jose	TG(16:0/18:1/24:1); TG(18:1/18:1/22:0)	TG(58:2)	1.71	0.03
Triacylglycerol	Jose	TG(16:1/18:2/18:2)_A	TG(52:5)	1.38	0.03
Triacylglycerol	Jose	TG(18:1/18:1/22:1)	TG(58:3)	2.04	0.02
Triacylglycerol	Jose	TG(18:2/18:3/24:1)	TG(60:6)	2.01	0.03
