# Supplementary material for: Influence of soil depth, irrigation, and plant genotype on the soil microbiome, metaphenome, and carbon chemistry
Source: mBio. 2023 Sep 20;14(5):e01758-23. doi: 10.1128/mbio.01758-23 (PMC10653930; doi:10.1128/mbio.01758-23)
Supplement: Table S5 — Differential gene abundances. [file mbio.01758-23-s0008.pdf]

Supp. Table 5. Differential gene abundance (Log2 Fold Change) of 16S (bacteria and archaea) and ITS (fungi) rRNA genes across experimental variables of depth (comparing top (0-5 cm) and bottom (48-100 cm) horizons), irrigation (comparing non-irrigated bare to irrigated bare soils), and cultivar (comparing irrigated bare soils to irrigated Alkar and Jose individually).

Variable	Kingdom	Phylum	Class	Order	Family	Genus	Species	Base Mean	FC	SE	P_{adj}
Depth	Archaea	Crenarchaeota	Nitrososphaeria	Nitrosopumilales	Nitrosopumilaceae	Candidatus Nitrosotenuis	Uncultured	9.66	21.77	2.95	1.95E-10
Depth	Bacteria	Proteobacteria	Gammaproteobacteria	Burkholderiales	Nitrosomonadaceae	MND1	Uncultured	16.53	22.52	2.95	4.63E-11
Depth	Bacteria	Nitrospirota	Nitrospiria	Nitrospirales	Nitrospiraceae	Nitrospira	Nitrospira enrichment	14.41	22.33	2.95	5.75E-11
Depth	Bacteria	Chloroflexi	Chloroflexia	Thermomicrobiales	JG30-KF-CM45	Uncultured	Uncultured	8.64	21.62	2.95	2.41E-10
Depth	Bacteria	Verrucomicrobiota	Verrucomicrobiae	Chthoniobacterales	Chthoniobacteraceae	Candidatus Udaeobacter	Uncultured	7.89	21.49	2.95	2.85E-10
Depth	Bacteria	Actinobacteriota	Actinobacteria	Micrococcales	Microbacteriaceae	Agromyces	Uncultured	8.91	-22.75	2.95	3.50E-11
Depth	Bacteria	Proteobacteria	Alphaproteobacteria	Sphingomonadales	Sphingomonadaceae	Sphingomonas	Uncultured	9.33	-22.78	2.95	3.50E-11
Depth	Fungi	Ascomycota	Dothideomycetes	Pleosporales	Pleosporaceae	Alternaria	Alternaria subcucurbitae	54.57	24.43	2.95	1.06E-13
Depth	Fungi	Ascomycota	Sordariomycetes	Hypocreales	Nectriaceae	Fusarium	Unidentified	50.11	23.79	2.95	1.78E-13
Depth	Fungi	Ascomycota	Dothideomycetes	Pleosporales	Pleosporaceae	Curvularia	Curvularia lunata	15.54	-23.40	2.96	4.83E-13
Depth	Fungi	Ascomycota	Dothideomycetes	Pleosporales	Phaeosphaeriaceae	Neosetophoma	Unidentified	22.16	-23.61	2.9	1.09E-13
Depth	Fungi	Mortierellomycota	Mortierellomycetes	Mortierellales	Mortierellaceae	Mortierella	Unidentified	29.46	-24.28	2.96	1.06E-13
Irrigation	Archaea	Crenarchaeota	Nitrososphaeria	Nitrososphaerales	Nitrososphaeraceae	Candidatus Nitrososphaera	Uncultured	18.52	7.79	2.31	1.47E-02
Irrigation	Archaea	Crenarchaeota	Nitrososphaeria	Nitrosopumilales	Nitrosopumilaceae	Candidatus Nitrosotenuis	Uncultured	50.61	7.53	1.93	2.70E-03
Irrigation	Archaea	Crenarchaeota	Nitrososphaeria	Nitrososphaerales	Nitrososphaeraceae	Uncultured	Uncultured	21.97	-22.73	2.91	2.52E-13
Irrigation	Bacteria	Actinobacteriota	Actinobacteria	Pseudonocardiales	Pseudonocardiaceae	Crossiella	Uncultured	21.36	23.94	2.81	7.20E-15
Irrigation	Bacteria	Actinobacteriota	Actinobacteria	Streptomycetales	Streptomycetaceae	Streptomyces	Uncultured	18.56	23.77	2.96	1.64E-13
Irrigation	Bacteria	Actinobacteriota	Actinobacteria	Micrococcales	Micrococcaceae	Arthrobacter	Uncultured	17.94	23.73	2.96	1.64E-13
Irrigation	Bacteria	Nitrospirota	Nitrospiria	Nitrospirales	Nitrospiraceae	Nitrospira	Nitrospira enrichment	17.40	23.67	2.96	1.64E-13

(Supp. Table 5 continued)

Variable	Kingdom	Phylum	Class	Order	Family	Genus	Species	Base Mean	FC	SE	P_{adj}
Irrigation	Bacteria	Acidobacteriota	Blastocatellia	Pyrinomonadales	Pyrinomonadaceae	RB41	Uncultured	17.03	23.61	2.96	1.64E-13
Irrigation	Bacteria	Actinobacteriota	Actinobacteria	Micrococcales	Micrococcaceae	Kocuria	Uncultured	15.01	23.48	2.96	2.04E-13
Irrigation	Bacteria	Myxococcota	Polyangia	Haliangiales	Haliangiaceae	Haliangium	Uncultured	14.12	23.40	2.96	2.11E-13
Irrigation	Bacteria	Actinobacteriota	Thermoleophilia	Gaiellales	Gaiellaceae	Gaiella	Uncultured	14.17	23.38	2.96	2.11E-13
Irrigation	Bacteria	Acidobacteriota	Thermoanaerobaculia	Thermoanaerobaculales	Thermoanaerobaculaceae	Uncultured	Uncultured	13.47	23.34	2.96	2.17E-13
Irrigation	Bacteria	Acidobacteriota	Vicinamibacteria	Vicinamibacterales	Uncultured	Uncultured	Uncultured	13.13	23.3	2.96	2.19E-13
Irrigation	Bacteria	Acidobacteriota	Subgroup 25	Uncultured	Uncultured	Uncultured	Uncultured	12.76	23.26	2.96	2.25E-13
Irrigation	Bacteria	Dependentiae	Babeliae	Babeliales	Vermiphilaceae	Uncultured	Uncultured	11.80	23.16	2.96	2.52E-13
Irrigation	Bacteria	Myxococcota	Polyangia	Blfdi19	Uncultured	Blfdi19	Uncultured	11.75	23.15	2.96	2.52E-13
Irrigation	Bacteria	Actinobacteriota	Thermoleophilia	Uncultured	Uncultured	Uncultured	Uncultured	10.10	22.92	2.96	3.57E-13
Irrigation	Bacteria	Acidobacteriota	Vicinamibacteria	Vicinamibacterales	Uncultured	Uncultured	Uncultured	11.94	21.10	2.96	3.37E-11
Irrigation	Bacteria	Proteobacteria	Gammaproteobacteria	Burkholderiales	Nitrosomonadaceae	MND1	Uncultured	39.07	8.87	1.73	9.86E-06
Irrigation	Bacteria	Proteobacteria	Gammaproteobacteria	Burkholderiales	Nitrosomonadaceae	MND1	Uncultured	32.65	8.61	1.93	2.41E-04
Irrigation	Bacteria	Acidobacteriota	Subgroup 25	Uncultured	Uncultured	Uncultured	Uncultured	28.21	8.40	2.57	1.98E-02
Irrigation	Bacteria	Planctomycetota	Pla4 lineage	Uncultured	Uncultured	Uncultured	Uncultured	27.44	8.36	2.58	2.07E-02
Irrigation	Bacteria	Bacteroidota	Bacteroidia	Flavobacteriales	Flavobacteriaceae	Salinimicrobium	Uncultured	24.94	8.22	2.58	2.51E-02
Irrigation	Bacteria	Actinobacteriota	Actinobacteria	Streptomycetales	Streptomycetaceae	Streptomyces	Uncultured	24.74	8.21	2.34	1.05E-02
Irrigation	Bacteria	Proteobacteria	Gammaproteobacteria	Burkholderiales	Nitrosomonadaceae	MND1	Uncultured	19.24	7.85	2.54	3.19E-02
Irrigation	Bacteria	Firmicutes	Bacilli	Alicyclobacillales	Alicyclobacillaceae	Tumebacillus	Uncultured	18.90	7.82	2.58	3.58E-02
Irrigation	Bacteria	Actinobacteriota	Actinobacteria	Streptomycetales	Streptomycetaceae	Streptomyces	Uncultured	18.45	7.78	2.31	1.47E-02
Irrigation	Bacteria	Bdellovibrionota	Oligoflexia	0319-6G20	Uncultured	Uncultured	Uncultured	15.45	7.53	1.9	2.12E-03
Irrigation	Bacteria	Gemmatimonadota	BD2-11 terrestrial group	Uncultured	Uncultured	Uncultured	Uncultured	13.39	7.32	2.52	4.91E-02

(Supp. Table 5 continued)

Variable	Kingdom	Phylum	Class	Order	Family	Genus	Species	Base Mean	FC	SE	P_{adj}
Irrigation	Bacteria	Patescibacteria	ABY1	Candidatus Magasanikbacteria	Uncultured	Uncultured	Uncultured	13.25	7.31	1.93	4.01E-03
Irrigation	Bacteria	Proteobacteria	NB1-j	Uncultured	Uncultured	Uncultured	Uncultured	11.85	7.15	2.11	1.44E-02
Irrigation	Bacteria	Chloroflexi	Anaerolineae	Anaerolineales	Anaerolineaceae	Uncultured	Uncultured	11.16	7.06	2.32	3.58E-02
Irrigation	Bacteria	Elusimicrobiota	Lineage IIa	Uncultured	Uncultured	Uncultured	Uncultured	11.00	7.04	2.09	1.47E-02
Irrigation	Bacteria	Gemmatimonadota	Gemmatimonadetes	Gemmatimonadales	Gemmatimonadaceae	Uncultured	Uncultured	8.97	6.74	2.07	1.98E-02
Irrigation	Bacteria	Bacteroidota	SJA-28	SJA-28	Uncultured	SJA-28	Uncultured	30.29	5.58	1.63	1.34E-02
Irrigation	Bacteria	Verrucomicrobiota	Verrucomicrobiae	Pedosphaerales	Pedosphaeraceae	Uncultured	Uncultured	25.77	-5.01	1.59	2.77E-02
Irrigation	Bacteria	Proteobacteria	Gammaproteobacteria	Acidiferrobacterales	Acidiferrobacteraceae	Sulfurifustis	Uncultured	8.60	-6.37	2.19	4.91E-02
Irrigation	Bacteria	Actinobacteriota	Actinobacteria	Streptosporangiales	Thermomonosporaceae	Actinomadura	Uncultured	8.93	-6.43	2.21	4.91E-02
Irrigation	Bacteria	Bacteroidota	Bacteroidia	Chitinophagales	Chitinophagaceae	Edaphobaculum	Uncultured	9.73	-6.55	1.68	2.71E-03
Irrigation	Bacteria	Planctomycetota	Planctomycetes	Gemmatales	Gemmataceae	Uncultured	Uncultured	10.10	-6.61	2.02	1.98E-02
Irrigation	Bacteria	Acidobacteriota	Vicinamibacteria	Vicinamibacterales	Uncultured	Uncultured	Uncultured	10.62	-6.68	1.85	7.41E-03
Irrigation	Bacteria	Verrucomicrobiota	Verrucomicrobiae	Chthoniobacterales	Chthoniobacteraceae	Chthoniobacter	Uncultured	11.45	-6.79	2.20	3.19E-02
Irrigation	Bacteria	Acidobacteriota	Vicinamibacteria	Vicinamibacterales	Vicinamibacteraceae	Uncultured	Uncultured	11.62	-6.81	1.85	5.81E-03
Irrigation	Bacteria	Actinobacteriota	Actinobacteria	Micromonosporales	Micromonosporaceae	Virgisporangium	Uncultured	13.21	-6.99	2.20	2.54E-02
Irrigation	Bacteria	Methylomirabilota	Methylomirabilia	Rokubacteriales	Rokubacteriales	Rokubacteriales	Uncultured	15.24	-7.20	2.42	4.18E-02
Irrigation	Bacteria	Bacteroidota	Bacteroidia	Chitinophagales	Chitinophagaceae	Edaphobaculum	Uncultured	16.25	-7.29	2.04	8.21E-03
Irrigation	Bacteria	Proteobacteria	Alphaproteobacteria	Rhizobiales	Rhizobiaceae	Rhizobiaceae	Uncultured	16.68	-7.33	2.46	4.18E-02
Irrigation	Bacteria	Bacteroidota	Bacteroidia	Cytophagales	Hymenobacteraceae	Adhaeribacter	Adhaeribacter sp.	17.99	-7.44	2.44	3.52E-02
Irrigation	Bacteria	Bacteroidota	Bacteroidia	Chitinophagales	Chitinophagaceae	Ferruginibacter	Uncultured	21.73	-7.71	2.25	1.34E-02
Irrigation	Bacteria	Proteobacteria	Alphaproteobacteria	Rhizobiales	Xanthobacteraceae	Uncultured	Uncultured	22.49	-7.76	2.67	4.91E-02
Irrigation	Bacteria	Proteobacteria	Gammaproteobacteria	Steroidobacterales	Steroidobacteraceae	Steroidobacter	Uncultured	38.57	-8.54	2.50	1.34E-02

(Supp. Table 5 continued)

Variable	Kingdom	Phylum	Class	Order	Family	Genus	Species	Base Mean	FC	SE	P_{adj}
Irrigation	Bacteria	Proteobacteria	Gammaproteobacteria	Steroidobacterales	Steroidobacteraceae	Steroidobacter	Uncultured	95.45	-9.85	1.60	2.32E-08
Irrigation	Bacteria	Gemmatimonadota	Gemmatimonadetes	Gemmatimonadales	Gemmatimonadaceae	Uncultured	Uncultured	14.86	-22.19	2.88	4.59E-13
Irrigation	Bacteria	Verrucomicrobiota	Verrucomicrobiae	Chthoniobacterales	Chthoniobacteraceae	Candidatus Udaeobacter	Uncultured	16.64	-22.35	2.88	3.45E-13
Irrigation	Bacteria	Proteobacteria	Gammaproteobacteria	Steroidobacterales	Steroidobacteraceae	Uncultured	Uncultured	17.27	-22.4	2.89	3.45E-13
Irrigation	Bacteria	Proteobacteria	Alphaproteobacteria	Rhizobiales	Devosiaceae	Devosia	Uncultured	19.22	-22.55	2.91	3.45E-13
Irrigation	Bacteria	Nitrospirota	Nitrospiria	Nitrospirales	Nitrospiraceae	Nitrospira	Uncultured	22.63	-22.77	2.91	2.52E-13
Irrigation	Bacteria	Proteobacteria	Gammaproteobacteria	Burkholderiales	TRA3-20	Uncultured	Uncultured	36.76	-23.45	2.7	3.37E-15
Irrigation	Bacteria	Proteobacteria	Gammaproteobacteria	Burkholderiales	Nitrosomonadaceae	IS-44	Uncultured	45.35	-23.74	2.97	1.64E-13
Irrigation	Fungi	Unidentified	Unidentified	Unidentified	Unidentified	Unidentified	Unidentified	31.59	24.3	2.79	3.89E-16
Irrigation	Fungi	Mortierellomycota	Mortierellomycetes	Mortierellales	Mortierellaceae	Mortierella	Mortierella amoeboidea	17.38	23.46	2.97	9.76E-14
Irrigation	Fungi	Basidiomycota	Agaricomycetes	Agaricales	Agaricales	Agaricales	Unidentified	14.06	23.19	2.97	1.78E-13
Irrigation	Fungi	Ascomycota	Eurotiomycetes	Eurotiales	Aspergillaceae	Penicillium	Unidentified	12.78	23.07	2.97	2.19E-13
Irrigation	Fungi	Ascomycota	Sordariomycetes	Hypocreales	Hypocreaceae	Trichoderma	Unidentified	7.30	22.31	2.97	1.10E-12
Irrigation	Fungi	Mortierellomycota	Mortierellomycetes	Mortierellales	Mortierellaceae	Mortierella	Unidentified	72.35	9.60	2.57	1.98E-03
Irrigation	Fungi	Unidentified	Unidentified	Unidentified	Unidentified	Unidentified	Unidentified	56.92	9.26	2.13	2.04E-04
Irrigation	Fungi	Ascomycota	Dothideomycetes	Pleosporales	Didymosphaeriaceae	Pseudopithomyces	Pseudopithomyces rosae	28.07	8.24	2.06	7.69E-04
Irrigation	Fungi	Basidiomycota	Agaricomycetes	Agaricales	Psathyrellaceae	Coprinopsis	Coprinopsis asiaticiphlyctidospora	26.81	8.17	2.29	3.82E-03
Irrigation	Fungi	Blastocladiomycota	Blastocladiomycetes	Blastocladiales	Catenariaceae	Unidentified	Unidentified	18.91	7.67	2.76	4.94E-02
Irrigation	Fungi	Ascomycota	Eurotiomycetes	Onygenales	Onygenales Incertae sedis	Chrysosporium	Chrysosporium pseudomerdarium	6.84	6.20	2.22	4.94E-02
Irrigation	Fungi	Basidiomycota	Agaricomycetes	Agaricales	Entolomataceae	Clitopilus	Unidentified	483.14	-6.99	1.67	3.45E-04
Irrigation	Fungi	Basidiomycota	Agaricomycetes	Agaricales	Psathyrellaceae	Coprinopsis	Coprinopsis kubickae	18.21	-7.73	2.61	2.93E-02
Irrigation	Fungi	Basidiomycota	Agaricomycetes	Cantharellales	Ceratobasidiaceae	Thanatephorus	Thanatephorus cucumeris	26.09	-8.25	2.43	6.73E-03

(Supp. Table 5 continued)

Variable	Kingdom	Phylum	Class	Order	Family	Genus	Species	Base Mean	FC	SE	P_{adj}
Irrigation	Fungi	Glomeromycota	Glomeromycetes	Glomerales	Glomeraceae	Glomus	Unidentified	37.54	-8.78	2.26	1.18E-03
Irrigation	Fungi	Basidiomycota	Agaricomycetes	Thelephorales	Thelephoraceae	Unidentified	Unidentified	75.41	-9.78	2.26	2.04E-04
Irrigation	Fungi	Basidiomycota	Agaricomycetes	Agaricales	Entolomataceae	Entoloma	Unidentified	83.85	-9.94	2.33	2.62E-04
Irrigation	Fungi	Basidiomycota	Agaricomycetes	Cantharellales	Ceratobasidiaceae	Rhizoctonia	Rhizoctonia fusispora	9.96	-21.77	2.98	4.24E-12
Irrigation	Fungi	Basidiomycota	Agaricomycetes	Thelephorales	Thelephoraceae	Unidentified	Unidentified	11.19	-21.92	2.98	3.03E-12
Irrigation	Fungi	Basidiomycota	Agaricomycetes	Thelephorales	Unidentified	Unidentified	Unidentified	13.16	-22.15	2.98	1.80E-12
Irrigation	Fungi	Unidentified	Unidentified	Unidentified	Unidentified	Unidentified	Unidentified	14.64	-22.30	2.98	1.31E-12
Irrigation	Fungi	Mortierellomycota	Mortierellomycetes	Mortierellales	Mortierellaceae	Mortierella	Unidentified	15.68	-22.40	2.98	1.10E-12
Irrigation	Fungi	Basidiomycota	Agaricomycetes	Cantharellales	Ceratobasidiaceae	Unidentified	Unidentified	17.62	-22.56	2.98	8.37E-13
Irrigation	Fungi	Basidiomycota	Agaricomycetes	Thelephorales	Unidentified	Unidentified	Unidentified	19.21	-22.68	2.98	6.77E-13
Irrigation	Fungi	Basidiomycota	Agaricomycetes	Agaricales	Tricholomataceae	Melanoleuca	Melanoleuca microcephala	19.60	-22.70	2.98	6.77E-13
Irrigation	Fungi	Basidiomycota	Agaricomycetes	Agaricales	Bolbitiaceae	Conocybe	Conocybe confundens	25.82	-23.09	2.90	8.25E-14
Irrigation	Fungi	Basidiomycota	Agaricomycetes	Agaricales	Inocybaceae	Inocybe	Inocybe pararubens var. padjelantae	27.71	-23.19	2.88	5.03E-14
Irrigation	Fungi	Basidiomycota	Agaricomycetes	Thelephorales	Unidentified	Unidentified	Unidentified	31.25	-23.36	2.66	3.89E-16
Irrigation	Fungi	Ascomycota	Pezizomycetes	Pezizales	Pezizaceae	Iodophanus	Iodophanus testaceus	54.92	-23.39	2.93	7.67E-14
Irrigation	Fungi	Mortierellomycota	Mortierellomycetes	Mortierellales	Mortierellaceae	Mortierella	Unidentified	84.33	-24.75	2.97	6.70E-15
Alkar	Archaea	Crenarchaeota	Nitrososphaeria	Nitrosopumilales	Nitrosopumilaceae	Candidatus Nitrosotenuis	Uncultured	50.95	-9.22	1.96	9.62E-05
Alkar	Archaea	Crenarchaeota	Nitrososphaeria	Nitrosopumilales	Nitrosopumilaceae	Nitrosarchaeum	Uncultured	68.86	-9.65	2.25	6.47E-04
Alkar	Bacteria	Verrucomicrobiota	Chlamydiae	Chlamydiales	Parachlamydiaceae	Uncultured	Uncultured	24.52	23.76	2.96	4.50E-13
Alkar	Bacteria	Verrucomicrobiota	Verrucomicrobiae	Pedosphaerales	Pedosphaeraceae	Uncultured	Uncultured	21.56	23.58	2.96	4.50E-13
Alkar	Bacteria	Actinobacteriota	Thermoleophilia	Solirubrobacterales	Uncultured	Uncultured	Uncultured	19.00	23.41	2.96	5.82E-13
Alkar	Bacteria	Proteobacteria	Gammaproteobacteria	Acidiferrobacterales	Acidiferrobacteraceae	Sulfurifustis	Uncultured	16.38	23.21	2.96	8.42E-13

(Supp. Table 5 continued)

Variable	Kingdom	Phylum	Class	Order	Family	Genus	Species	Base Mean	FC	SE	P_{adj}
Alkar	Bacteria	Acidobacteriota	Blastocatellia	Blastocatellales	Blastocatellaceae	Uncultured	Uncultured	12.13	22.81	2.96	1.34E-12
Alkar	Bacteria	Bacteroidota	Bacteroidia	Cytophagales	Microscillaceae	Uncultured	Uncultured	11.59	22.73	2.96	1.42E-12
Alkar	Bacteria	Actinobacteriota	Actinobacteria	Streptomycetales	Streptomycetaceae	Streptomyces	Streptomyces	11.41	22.72	2.96	1.42E-12
Alkar	Bacteria	Acidobacteriota	Thermoanaerobaculia	Thermoanaerobaculales	Thermoanaerobaculaceae	Subgroup 10	Uncultured	10.58	22.62	2.96	1.61E-12
Alkar	Bacteria	Bacteroidota	Bacteroidia	Cytophagales	Microscillaceae	Uncultured	Uncultured	9.54	22.48	2.96	2.05E-12
Alkar	Bacteria	Acidobacteriota	Thermoanaerobaculia	Thermoanaerobaculales	Thermoanaerobaculaceae	Subgroup 10	Uncultured	19.36	22.48	2.96	2.05E-12
Alkar	Bacteria	Acidobacteriota	Blastocatellia	Pyrinomonadales	Pyrinomonadaceae	RB41	Uncultured	8.47	22.32	2.96	2.42E-12
Alkar	Bacteria	Cyanobacteria	Cyanobacteriia	Phormidesmiales	Nodosilineaceae	Nodosilinea PCC-7104	Nodosilinea sp.	7.19	21.75	2.96	8.83E-12
Alkar	Bacteria	Desulfobacterota	Desulfuromonadia	Geobacterales	Geobacteraceae	Citrifermentans	Uncultured	18.98	21.42	2.96	1.89E-11
Alkar	Bacteria	Proteobacteria	Alphaproteobacteria	Dongiales	Dongiaceae	Dongia	Uncultured	21.33	7.81	2.42	4.18E-02
Alkar	Bacteria	Verrucomicrobiota	Verrucomicrobiae	Opitutales	Opitutaceae	Opitutus	Uncultured	19.60	7.69	2.41	4.68E-02
Alkar	Bacteria	Patescibacteria	ABY1	Candidatus Magasanikbacteria	Unidentified	Unidentified	Uncultured	13.23	-7.27	1.95	6.50E-03
Alkar	Bacteria	Bacteroidota	Bacteroidia	Flavobacteriales	Flavobacteriaceae	Salinimicrobium	Uncultured	25.93	-8.24	2.61	4.97E-02
Alkar	Bacteria	Myxococcota	Polyangia	Haliangiales	Haliangiaceae	Haliangium	Uncultured	14.17	-20.47	2.95	1.68E-10
Alkar	Bacteria	Acidobacteriota	Blastocatellia	Blastocatellales	Blastocatellaceae	Aridibacter	Aridibacter	9.51	-21.94	2.96	5.02E-12
Alkar	Bacteria	Actinobacteriota	Thermoleophilia	Uncultured	Uncultured	Uncultured	Uncultured	9.64	-21.95	2.96	5.00E-12
Alkar	Bacteria	Proteobacteria	Gammaproteobacteria	R7C24	Uncultured	Uncultured	Uncultured	11.36	-22.18	2.95	2.93E-12
Alkar	Bacteria	Dependentiae	Babeliae	Babeliales	Vermiphilaceae	Uncultured	Uncultured	12.18	-22.28	2.95	2.42E-12
Alkar	Bacteria	Actinobacteriota	Thermoleophilia	Gaiellales	Uncultured	Uncultured	Uncultured	12.93	-22.36	2.95	2.08E-12
Alkar	Bacteria	Acidobacteriota	Vicinamibacteria	Vicinamibacterales	Uncultured	Uncultured	Uncultured	13.18	-22.39	2.95	2.05E-12
Alkar	Bacteria	Acidobacteriota	Thermoanaerobaculia	Thermoanaerobaculales	Thermoanaerobaculaceae	Subgroup 10	Uncultured	13.30	-22.4	2.95	2.05E-12
Alkar	Bacteria	Actinobacteriota	Actinobacteria	Micrococcales	Micrococcaceae	Kocuria	Uncultured	15.71	-22.64	2.95	1.45E-12

(Supp. Table 5 continued)

Variable	Kingdom	Phylum	Class	Order	Family	Genus	Species	Base Mean	FC	SE	P_{adj}
Alkar	Bacteria	Actinobacteriota	Actinobacteria	Streptomycetales	Streptomycetaceae	Streptomyces	Uncultured	17.29	-22.77	2.95	1.34E-12
Alkar	Bacteria	Nitrospirota	Nitrospiria	Nitrospirales	Nitrospiraceae	Nitrospira	Uncultured	17.80	-22.81	2.95	1.34E-12
Alkar	Bacteria	Actinobacteriota	Actinobacteria	Micrococcales	Micrococcaceae	Arthrobacter	Uncultured	18.68	-22.88	2.95	1.33E-12
Alkar	Bacteria	Actinobacteriota	Actinobacteria	Streptomycetales	Streptomycetaceae	Streptomyces	Uncultured	18.75	-22.88	2.95	1.33E-12
Alkar	Bacteria	Verrucomicrobiota	Verrucomicrobiae	Chthoniobacterales	Chthoniobacteraceae	Candidatus Udaeobacter	Uncultured	31.19	-23.59	2.95	4.50E-13
Alkar	Bacteria	Nitrospirota	Nitrospiria	Nitrospirales	Nitrospiraceae	Nitrospira	Nitrospirae bacterium	39.35	-23.91	2.95	4.50E-13
Alkar	Fungi	Ascomycota	Pezizomycetes	Pezizales	Pyronemataceae	Pulvinula	Unidentified	120.6	25.97	2.6	4.42E-21
Alkar	Fungi	Ascomycota	Sordariomycetes	Hypocreales	Nectriaceae	Fusarium	Unidentified	87.39	25.53	2.99	1.97E-15
Alkar	Fungi	Ascomycota	Sordariomycetes	Hypocreales	Hypocreales	Hypocreales	Unidentified	30.39	24.09	2.99	5.67E-14
Alkar	Fungi	Mortierellomycota	Mortierellomycetes	Mortierellales	Mortierellaceae	Mortierella	Unidentified	16.92	23.29	2.99	3.92E-13
Alkar	Fungi	Unidentified	Unidentified	Unidentified	Unidentified	Unidentified	Unidentified	14.86	23.12	3.00	5.13E-13
Alkar	Fungi	Basidiomycota	Agaricomycetes	Thelephorales	Thelephoraceae	Unidentified	Unidentified	12.28	22.82	3.00	9.56E-13
Alkar	Fungi	Ascomycota	Pezizomycetes	Pezizales	Unidentified	Unidentified	Unidentified	10.76	22.21	3.00	3.58E-12
Alkar	Fungi	Basidiomycota	Agaricomycetes	Cantharellales	Ceratobasidiaceae	Thanatephorus	Thanatephorus cucumeris	78.25	9.87	2.00	1.91E-05
Alkar	Fungi	Ascomycota	Dothideomycetes	Pleosporales	Lophiostomataceae	Unidentified	Unidentified	46.59	9.13	2.53	5.57E-03
Alkar	Fungi	Glomeromycota	Glomeromycetes	Glomerales	Glomeraceae	Glomus	Unidentified	29.54	8.47	2.33	5.10E-03
Alkar	Fungi	Unidentified	Unidentified	Unidentified	Unidentified	Unidentified	Unidentified	28.83	8.43	2.29	4.73E-03
Alkar	Fungi	Mortierellomycota	Mortierellomycetes	Mortierellales	Mortierellaceae	Mortierella	Mortierella polygonia	22.95	8.10	2.73	4.87E-02
Alkar	Fungi	Basidiomycota	Agaricomycetes	Agaricales	Entolomataceae	Entoloma	Unidentified	20.90	7.97	2.13	4.05E-03
Alkar	Fungi	Basidiomycota	Agaricomycetes	Agaricales	Unidentified	Unidentified	Unidentified	8.60	-21.95	2.98	4.23E-12
Alkar	Fungi	Ascomycota	Sordariomycetes	Hypocreales	Nectriaceae	Fusarium	Unidentified	13.51	-22.58	2.98	1.05E-12
Alkar	Fungi	Unidentified	Unidentified	Unidentified	Unidentified	Unidentified	Unidentified	28.47	-23.56	2.81	4.84E-15
Jose	Archaea	Crenarchaeota	Nitrososphaeria	Nitrososphaerales	Nitrososphaeraceae	Candidatus Nitrososphaera	Uncultured	18.98	-7.93	2.33	3.45E-02
Jose	Archaea	Crenarchaeota	Nitrososphaeria	Nitrosopumilales	Nitrosopumilaceae	Candidatus Nitrosotenuis	Uncultured	49.10	-9.30	1.95	1.44E-04

(Supp. Table 5 continued)

Variable	Kingdom	Phylum	Class	Order	Family	Genus	Species	Base Mean	FC	SE	P_{adj}
Jose	Archaea	Crenarchaeota	Nitrososphaeria	Nitrosopumilales	Nitrosopumilaceae	Nitrosarchaeum	Uncultured	66.54	-9.74	2.23	9.51E-04
Jose	Bacteria	Proteobacteria	Gammaproteobacteria	Pseudomonadales	Pseudomonadaceae	Pseudomonas	Uncultured	15.91	23.07	2.97	2.44E-12
Jose	Bacteria	Proteobacteria	Alphaproteobacteria	Rhizobiales	Xanthobacteraceae	Uncultured	Uncultured	26.09	8.00	2.06	6.20E-03
Jose	Bacteria	Firmicutes	Bacilli	Bacillales	Bacillaceae	Bacillus	Bacillus funiculus	17.42	7.42	2.25	4.72E-02
Jose	Bacteria	Myxococcota	Polyangia	Haliangiales	Haliangiaceae	Haliangium	Uncultured	15.02	7.21	2.05	2.42E-02
Jose	Bacteria	Verrucomicrobiota	Verrucomicrobiae	Verrucomicrobiales	Rubritaleaceae	Luteolibacter	Uncultured	11.42	6.81	1.70	4.05E-03
Jose	Bacteria	Verrucomicrobiota	Chlamydiae	Chlamydiales	Uncultured	Uncultured	Uncultured	9.36	-6.91	2.10	4.72E-02
Jose	Bacteria	Dependentiae	Babeliae	Babeliales	Vermiphilaceae	Uncultured	Uncultured	12.35	-22.55	2.96	2.44E-12
Jose	Bacteria	Acidobacteriota	Thermoanaerobaculia	Thermoanaerobaculales	Thermoanaerobaculaceae	Subgroup 10	Uncultured	12.33	-22.55	2.96	2.44E-12
Jose	Bacteria	Myxococcota	Polyangia	Haliangiales	Haliangiaceae	Haliangium	Uncultured	12.81	-22.61	2.96	2.44E-12
Jose	Bacteria	Verrucomicrobiota	Verrucomicrobiae	Pedosphaerales	Pedosphaeraceae	Uncultured	Uncultured	13.29	-22.66	2.96	2.44E-12
Jose	Bacteria	Actinobacteriota	Actinobacteria	Micrococcales	Micrococcaceae	Kocuria	Uncultured	15.99	-22.79	2.96	2.44E-12
Jose	Bacteria	Actinobacteriota	Actinobacteria	Streptomycetales	Streptomycetaceae	Streptomyces	Uncultured	14.85	-22.81	2.96	2.44E-12
Jose	Bacteria	Actinobacteriota	Actinobacteria	Micrococcales	Micrococcaceae	Arthrobacter	Uncultured	19.00	-23.16	2.96	2.44E-12
Jose	Fungi	Ascomycota	Pezizomycetes	Pezizales	Unidentified	Unidentified	Unidentified	49.85	24.51	2.60	1.25E-18
Jose	Fungi	Unidentified	Unidentified	Unidentified	Unidentified	Unidentified	Unidentified	32.54	23.93	2.72	1.83E-16
Jose	Fungi	Ascomycota	Sordariomycetes	Hypocreales	Hypocreales Incertae sedis	Sesquicillium	Sesquicillium microsporum	24.58	23.53	2.74	8.06E-16
Jose	Fungi	Unidentified	Unidentified	Unidentified	Unidentified	Unidentified	Unidentified	16.65	23.01	2.99	6.91E-13
Jose	Fungi	Ascomycota	Sordariomycetes	Sordariales	Chaetomiaceae	Humicola	Humicola nigrescens	8.39	22.08	3.00	5.10E-12
Jose	Fungi	Ascomycota	Pezizomycetes	Pezizales	Pyronemataceae	Pulvinula	Unidentified	32.68	8.30	2.37	8.38E-03
Jose	Fungi	Glomeromycota	Glomeromycetes	Glomerales	Glomeraceae	Glomus	Unidentified	17.62	7.41	1.94	2.64E-03
Jose	Fungi	Ascomycota	Leotiomycetes	Helotiales	Hyaloscyphaceae	Unidentified	Unidentified	298.86	7.07	1.44	2.41E-05
Jose	Fungi	Unidentified	Unidentified	Unidentified	Unidentified	Unidentified	Unidentified	16.58	6.67	2.26	4.98E-02

(Supp. Table 5 continued)

Variable	Kingdom	Phylum	Class	Order	Family	Genus	Species	Base Mean	FC	SE	P_{adj}
Jose	Fungi	Ascomycota	Dothideomycetes	Pleosporales	Didymosphaeriaceae	Laburnicola	Unidentified	86.19	-6.79	2.11	2.19E-02
Jose	Fungi	Blastocladiomycota	Blastocladiomycetes	Blastocladiales	Catenariaceae	Unidentified	Unidentified	42.83	-8.93	2.34	2.64E-03
Jose	Fungi	Mortierellomycota	Mortierellomycetes	Mortierellales	Mortierellaceae	Mortierella	Unidentified	83.74	-9.90	2.59	2.64E-03
Jose	Fungi	Basidiomycota	Agaricomycetes	Agaricales	Unidentified	Unidentified	Unidentified	6.56	-21.73	2.98	7.79E-12
Jose	Fungi	Basidiomycota	Basidiomycota	Basidiomycota	Unidentified	Unidentified	Unidentified	11.47	-22.51	2.98	1.31E-12
Jose	Fungi	Ascomycota	Sordariomycetes	Hypocreales	Nectriaceae	Fusarium	Unidentified	12.70	-22.65	2.98	1.05E-12
Jose	Fungi	Unidentified	Unidentified	Unidentified	Unidentified	Unidentified	Unidentified	48.21	-22.69	2.85	8.48E-14
Jose	Fungi	Unidentified	Unidentified	Unidentified	Unidentified	Unidentified	Unidentified	73.51	-24.33	2.86	1.25E-15
