# Supplementary material for: Influence of soil depth, irrigation, and plant genotype on the soil microbiome, metaphenome, and carbon chemistry
Source: mBio. 2023 Sep 20;14(5):e01758-23. doi: 10.1128/mbio.01758-23 (PMC10653930; doi:10.1128/mbio.01758-23)
Supplement: Table S6 — Common proteins. [file mbio.01758-23-s0009.pdf]

Supp. Table 6. Most common 50 proteins observed in n (number of samples)

**Protein ID**	**KO**	**EC**	**n**
Polar amino acid ABC transport	K02030		40
L-amino acid ABC transport	K09969		44
Alpha-glucoside ABC transport	K10232		43
Ribose ABC transport	K10439		43
Simple sugar ABC transport	K02058		42
Glucosylceramidase GBA	K01201	3.2.1.45	43
Histone H3	K11253		46
Histone H4	K11254		46
Plant pathogen enlong./interact.	K02358		42
Heat shock	K03283		40
chaperonin GroEL	K04077		46
Pilus assembly	K02650		43
Iron complex receptor	K02014		42
Superoxide dismutase, Fe-Mn	K04564		40
F-type ATPase, prokaryotes	K02112	7.1.2.2	42
F-type ATPase, prokaryotes	K02111	7.1.2.2	40
F-type ATPase, prokaryotes	K02110	7.1.2.2	41
Multiple sugar ABC transport, ATP-binding	K10112	7.5.2.-	38
Methane metabolism hydrolase Fae	K10713	4.2.1.147	42
Alcohol Dehydrogenase exaA	K00114	1.1.2.8	44
Glyceraldehyde Dehydrogenase GAPDH	K00134	1.2.1.12	40
Mineral/organic ion NitT/TauT family transport	K02051		42
BCAA ABC transport	K01999		45
Oligopeptide transport	K15580		42
Peptide-Ni ABC transport	K02035		44
Sugars and lipid ABC transport	K02027		43
Fructose ABC transport	K10552		42
D-xylose ABC transport	K10543		39
Membrane protein ABC transport	K07335		39
osmY	K04065		41
OmpA-OmpF porin	K03286		43
Omp31	K16079		45
Formamidase	K01455	3.5.1.49	42
Nitrite reductase	K00368	1.7.2.1	43
Urease subunit gamma ureA	K01430	3.5.1.5	42
Urease subunit gamma/beta UreAB	K14048	3.5.1.5	42
Urease subunit alpha ureC	K01428	3.5.1.5	39
DNA-directed RNA polymerase subunit alpha	K03040	2.7.7.6	39
Bacterioferritin bfr	K03594	1.16.3.1	40
Tubulin	K07374		42
Dodecin in prokaryotes	K09165		42
Peptidoglycan lipoprotein	K03640		40
DNA binding protein	K03530		40
GTP-binding nuclear protein RAN	K07936		39
Ribosomal protein	K02935		40
Outer membrane protein	K08720		40
14-3-3 protein YWHAE	K06630		39
Glycerol kinase glpK	K00864	2.7.1.30	39
Streptogrisin B sprB	K18545	3.4.21.81	40
Streptogrisin D sprD	K18547	3.4.21.-	38
